# Inhibition of group-I metabotropic glutamate receptors protects against prion toxicity

**DOI:** 10.1371/journal.ppat.1006733

**Published:** 2017-11-27

**Authors:** Despoina Goniotaki, Asvin K. K. Lakkaraju, Amulya N. Shrivastava, Pamela Bakirci, Silvia Sorce, Assunta Senatore, Rajlakshmi Marpakwar, Simone Hornemann, Fabrizio Gasparini, Antoine Triller, Adriano Aguzzi

**Affiliations:** 1 Institute of Neuropathology, University of Zurich, Zurich, Switzerland; 2 École Normale Supérieure, Institut de Biologie de l'ENS (IBENS) INSERM CNRS PSL Research University, Paris, France; 3 Paris-Saclay Institute of Neuroscience, CNRS, Gif-sur-Yvette, France; 4 Novartis Institutes for BioMedical Research, Basel, Switzerland; University of Western Ontario, CANADA

## Abstract

Prion infections cause inexorable, progressive neurological dysfunction and neurodegeneration. Expression of the cellular prion protein PrP^C^ is required for toxicity, suggesting the existence of deleterious PrP^C^-dependent signaling cascades. Because group-I metabotropic glutamate receptors (mGluR1 and mGluR5) can form complexes with the cellular prion protein (PrP^C^), we investigated the impact of mGluR1 and mGluR5 inhibition on prion toxicity *ex vivo* and *in vivo*. We found that pharmacological inhibition of mGluR1 and mGluR5 antagonized dose-dependently the neurotoxicity triggered by prion infection and by prion-mimetic anti-PrP^C^ antibodies in organotypic brain slices. Prion-mimetic antibodies increased mGluR5 clustering around dendritic spines, mimicking the toxicity of Aβ oligomers. Oral treatment with the mGluR5 inhibitor, MPEP, delayed the onset of motor deficits and moderately prolonged survival of prion-infected mice. Although group-I mGluR inhibition was not curative, these results suggest that it may alleviate the neurological dysfunctions induced by prion diseases.

## Author summary

Prion diseases are a result of ordered accumulation of the misfolded conformer of cellular prion protein (PrP^C^), a GPI anchored protein expressed on the cell surface. Similar pathogenetic principles operate in several other neurodegenerative diseases. Currently no disease-modifying therapies exist and the situation is compounded by a dearth of validated therapeutic targets. In our present study, we have discovered that genetic ablation, or pharmacological inhibition, of group-I (i.e. activating) metabotropic glutamate receptors is beneficial against prion neurotoxicity in vitro and in vivo. Mice treated with these inhibitors exhibited impressive suppression of neurological signs and a delayed onset of the symptoms. These results further suggest that activation of these metabotropic glutamate receptors is a downstream event of prion replication and targeting these receptors could be a therapeutic option to alleviate the neurological symptoms, thereby ameliorating the quality of life in patients having prion infection.

## Introduction

The decisive event in the pathogenesis of prion diseases is the conversion of the normal cellular prion protein (PrP^C^) into an aggregated conformational variant called PrP^Sc^ [1]. Expression of PrP^C^ at the cell surface is not only required for the self-propagation of prions, but also for mediating the toxicity induced by PrP^Sc^ [2], a process that results in endoplasmic reticulum (ER) stress and ultimately in impaired protein translation [3]. But how can PrP^C^, an extracellular GPI-linked protein, initiate intracellular central nervous system (CNS) toxicity? Most likely this process requires mediation by transmembrane constituents. Indeed PrP^C^ has been shown to interact with transmembrane signal-transducing proteins [4] and disturbing these interactions might lead to the neurotoxicity seen in prion diseases [5].

Among the proteins interacting with PrP^C^ are glutamate receptors [6]. N-methyl-D-aspartate receptors (NMDAR) are crucial regulators of glutamatergic transmission, and loss of both synapses and neurons has been attributed to inappropriate NMDAR activation [7, 8]. Metabotropic glutamate receptors (mGluRs) may also play a role in prion diseases. Changes in mGluR1, leading to reduced expression levels of phospholipases, were observed in the cerebral cortex of Creutzfeldt-Jakob disease (CJD) patients [9]. Also, impairment of the mGluR1/1-phosphatidylinositol 4,5-bisphosphate phosphodiesterase 1 (PLC1)/protein kinase C (PKC) signaling pathway has been observed in a murine model of BSE. Abnormal mGluR1 signaling correlated with PrP^Sc^ deposition, histological changes, and clinical scores [10].

A role for group-I mGluRs is emerging in a multitude of CNS disorders including Fragile X syndrome, ischemia, multiple sclerosis, amyotrophic lateral sclerosis, Huntington’s, and Parkinson’s disease [11–18]. In Alzheimer’s disease (AD), PrP^C^ and mGluR5 may directly contribute to disease manifestation and toxicity of amyloid-β (Aβ) aggregates. Aβ oligomers can bind to PrP^C^ at the cell surface [19] and form complexes that contain mGluR5 [20]. In a mouse model of Aβ deposition, cognitive decline and synaptic alterations were rescued by mGluR5 inhibition [21]. Furthermore, PrP^C^-mGluR5 coupling is involved in Aβ-mediated inhibition of LTP and Aβ-facilitated LTD *in vivo* [22], and genetic ablation of mGluR5 reverses disease-related memory deficits in a murine model of AD (APPswe/PS1ΔE9) [23]. In another study, exposure of cortical APPswe/PS1ΔE9 neuronal cultures to Aβ oligomers upregulated mGluR1 and PrP^C^ α-cleavage, whereas activation of group-I mGluRs increased PrP^C^ shedding from the membrane [24]. In primary hippocampal neurons, membrane-bound Aβ oligomers induce toxicity by promoting clustering of mGluR5 in synapses, resulting in elevated intracellular calcium and synaptic failure [25]. All these studies suggest an involvement of group-I mGluRs in the pathogenesis of AD. On the other hand, others have reported that neither PrP^C^ ablation nor overexpression had any effect on neurotoxicity in AD models [26–29]. As a possible explanation for these discrepancies, it has been suggested that only a limited oligomeric fraction of Aβ [30] interacts with mGluR5 [31].

Here we focused on the role of group-I mGluR-PrP^C^ interaction in prion disease. We found that toxic prion-mimetic compounds increased mGluR5 clustering and accumulation at dendritic heads, close to the synaptic source of glutamate. Moreover, pharmacological inhibition of mGluR1 and mGluR5, as well as genetic ablation of the *Grm5* gene encoding mGluR5, protected organotypic slice cultures against the toxicity of prions and of prion-mimetic compounds. Finally, pharmacological inhibition of mGluR5 improved the neurological status and, to some extent, the survival of prion-infected mice.

## Results

### Group-I mGluRs antagonists rescue prion-induced neurotoxicity in organotypic slices

Cerebellar and hippocampal organotypic cultured slices (COCS and HOCS, respectively) [32, 33] prepared from PrP^C^ overexpressing *tg*a*20* mice [34] can be infected with the Rocky Mountain Laboratory (RML) strain of prions and undergo neurodegeneration after ca. 5 weeks [32]. The time course and extent of neurodegeneration can be measured by morphometric assessment of the area of the cerebellar granule cell layer (CGL) immunoreactive to antibodies against the neuronal NeuN antigen.

We inoculated COCS and HOCS with brain homogenate from CD1 mice that had been infected with RML prions (passage #6, henceforth called RML6). For control, slices were inoculated with non-infectious brain homogenate (NBH) derived from healthy CD1 mice. Starting at 21 days post infection, slices were treated with a range of concentrations of either N-cyclohexyl-6-N-methylthiazolo[3,2-a]benzimidazole-2-carboxamide (YM202074) [35], 2-methyl-6-(phenylethynyl)-pyridine (MPEP) [36] or Mavoglurant (AFQ056) [37] which specifically inhibit mGluR1 and mGluR5, respectively.

**Fig 1:**
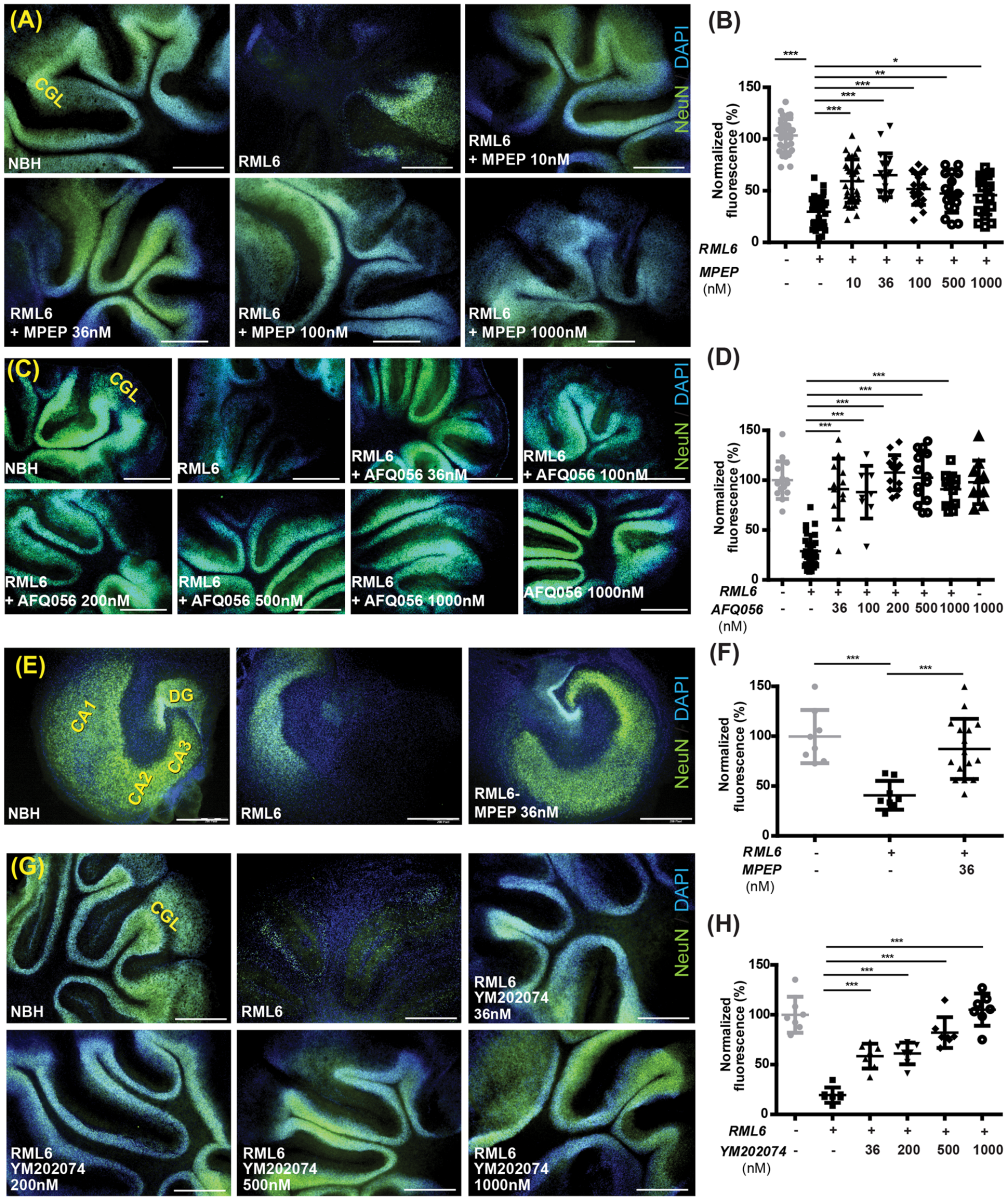
mGluR1/5 inhibition rescues prion neurotoxicity in organotypic slice cultures. **(A-B)** Treatment with the mGluR5 inhibitor (MPEP) rescued neurodegeneration in *tg*a*20* RML6-treated COCS. **(A)** Fluorescence micrographs of *tga20* COCS. RML6-induced ablation of the cerebellar granular layer (CGL) was significantly ameliorated by the mGluR5 inhibitor, MPEP. All scale bars: 500μm. **(B)** NeuN coverage in *tg*a*20* COCS exposed to RML6 or NBH and treated with MPEP at 21–45 days post inoculation (dpi), expressed as percentage of NBH samples. Each dot represents a pool of 4–10 slices cultured in the same well. Data points are mean ± s.d.; one-way ANOVA followed by Dunnett’s post-hoc test. **(C-D)** Treatment with the mGluR5 inhibitor AFQ056 (mavoglurant) also rescued neurodegeneration in *tg*a*20* RML6-treated COCS (experimental conditions as in panels A-B). **(E-F)** Treatment with the mGluR5 inhibitor (MPEP) rescued neurodegeneration in *tg*a*20* RML6-treated HOCS. **(E)** Fluorescence micrographs of *tga20* HOCS, showing ablation of hippocampal neurons induced by RML6 infection (middle), that is significantly ameliorated by addition of the IC50 concentration of MPEP (36nM, 21–45 dpi, right). **(F)** Morphometry of the experiment shown in panel E. **(G)** Treatment with the mGluR1 inhibitor (YM202074) rescued neurodegeneration in *tg*a*20* RML6-treated COCS. Experimental conditions were the same as in the panels above. **(H)** Morphometry of the experiment shown in panel G; *: P < 0.05, **: P < 0.01, ***: P < 0.001; For **(A)**, **(C)**, **(E)** and **(G)** panels: Scale bar is 500μm.

MPEP, AFQ056 and YM202074 prevented CGL loss in COCS at concentrations as low as 10 nM (Fig 1A and 1B) and 36 nM (Fig 1C, 1D, 1G and 1H), respectively. The protective effect of YM202074 and MPEP was further confirmed in wild-type slices (S1A and S1B Fig). Extremely high MPEP concentrations (3–10 μM) were not intrinsically toxic (S1C Fig) as previously reported [36], but failed to protect against prion toxicity in *tga*20 mice (S1C and S1D Fig). Also in HOCS, prepared from 4–6 days old *tg*a*20* mice, MPEP significantly suppressed neuronal loss after prion infection at concentrations as low as 36 nM (Fig 1E and 1F).

### MPEP alleviates the clinical signs of prion disease in mice

**Fig 2:**
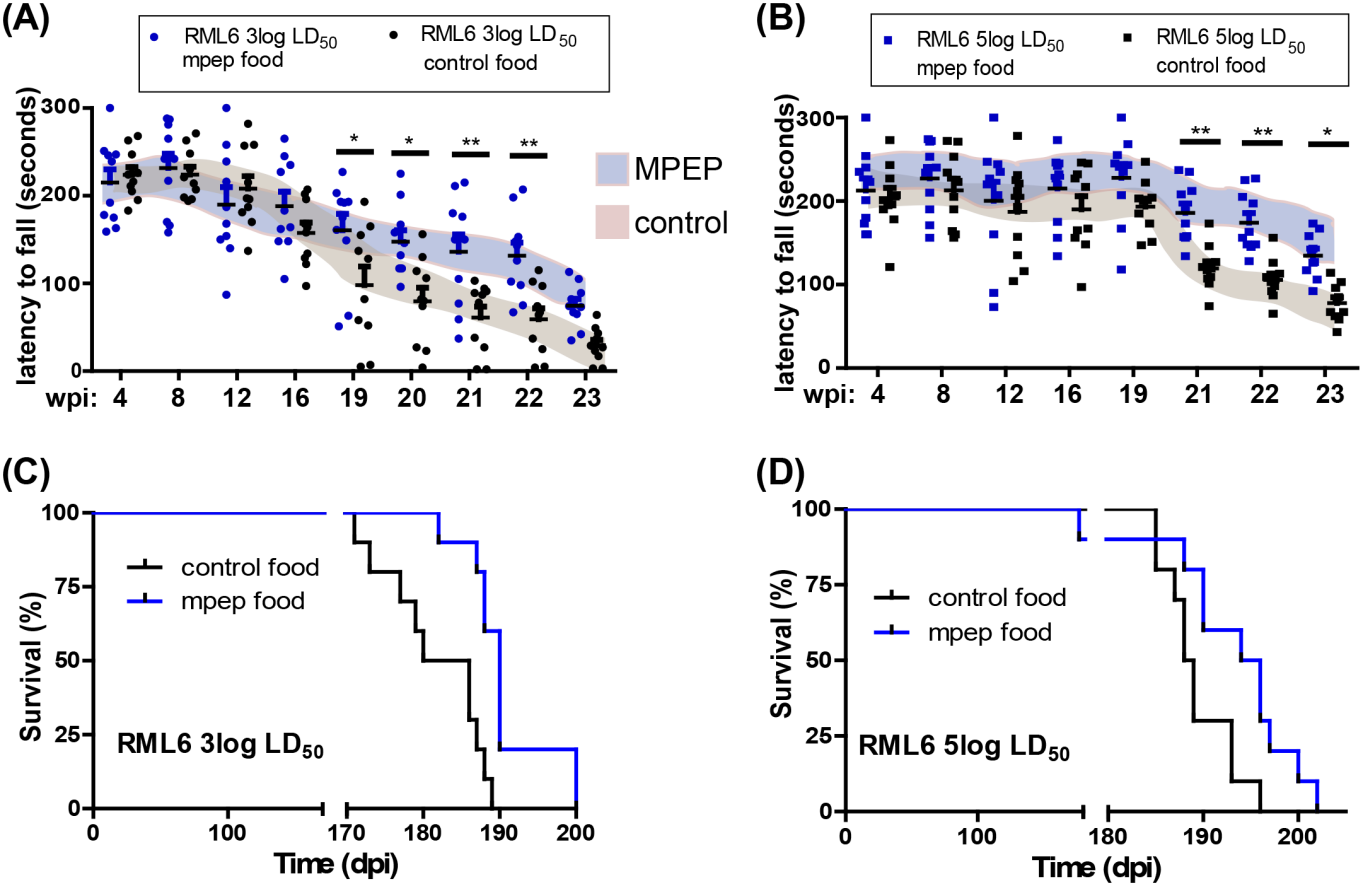
mGluR5 inhibition delays prion disease in wild-type mice. **(A-B)** MPEP improves motor performance in mouse models of prion disease. Motor abilities of MPEP-treated and control C57BL/6J males were assessed by rotarod after i.c. innoculation with 3 log LD_50_ **(A)** and 5 log LD_50_ **(B)** units of RML6 prions. Dot plots: latency to fall (seconds). Each dot corresponds to a mouse. Two-way ANOVA per each time point revealed a significant difference between MPEP treated and MPEP untreated groups at 19-22wpi (*: P<0.05 and **: P<0.01) for mice injected with 3 log LD_50_ RML6 units and at 21-23wpi (*: P<0.05 and **: P<0.001) for mice injected with 5 log LD_50_ RML6 units respectively, n = 10 mice per group. Shaded areas represent standard deviations. **(C-D)** mGluR5 inhibition (MPEP treatment) significantly prolonged survival in mouse models of prion disease. Survival curves of MPEP treated and MPEP untreated C57BL/6J males, inoculated i.c. with 3 log LD_50_ and 5 log LD_50_ units of RML6 prions respectively. **(C)** Mice inoculated with 3 log LD_50_ RML6 units: MPEP untreated group, n = 10, median incubation time 183 days post inoculation (dpi). MPEP treated group, n = 10, median incubation time 190 dpi; P = 0.0008; log-rank test. **(D)** Mice inoculated with 5 log LD_50_ RML6 units: MPEP untreated group, n = 10, median incubation time: 188.5 dpi, P = 0.0008; MPEP treated group, n = 10, median incubation time: 195dpi, P = 0.0231; log-rank test.

The beneficial effects of mGluR5 inhibition *ex vivo* encouraged us to assess whether MPEP can potentially rescue prion pathogenesis *in vivo*. C57BL/6J male mice were inoculated intracerebrally with 3 or 5 log LD_50_ units of RML6 prions as described [38] and chronically treated with MPEP. Control mice were inoculated with NBH. In order to record the neurological deficits associated with prion disease, we utilized the rotarod behavioral test which measures a combination of motor performance, coordination and balance [39]. Rotarod performance was similar in RML6- and NBH-inoculated mice until 18 weeks following prion inoculation. Starting from 19 weeks post inoculation, mice receiving control food showed a progressive decline in rotarod performance. The performance of MPEP-treated mice declined, but less rapidly. This improvement was lasting and detectable until the very late stages of the disease (22–23 weeks post inoculation; Fig 2A and 2B), suggesting that the progression of the disease was delayed by MPEP.

At very late time points, the general health status of all mice deteriorated to an extent that made it impossible to accurately measure their rotarod performance and eventually required euthanasia. Nevertheless, MPEP-treated mice showed a modest, though significant, prolongation of survival (Fig 2C and 2D). The median survival for untreated vs MPEP-treated RML6-inoculated C57BL/6J mice was, respectively, 183 vs 190 days post inoculation (dpi) after injection with 3 log LD_50_ units of prions and 188 vs 195 dpi after inoculation with 5 log LD_50_ units (P = 0.0008 and 0.0231 respectively; log-rank test). Control mice injected with NBH and treated with MPEP exhibited stable rotarod performance during the entire test period, up to 23 weeks post-injection (S2A Fig). No significant changes in average food and water consumption were observed between control and treatment groups during the experiment (S2B Fig). To determine the exposure of the brain to MPEP, mice treated with control and MPEP food were sacrificed at two time points, corresponding to the active and the inactive phase of the mice across the circadian circle. The average brain-to-blood ratio for the MPEP concentration was around 1, indicating good brain penetration of MPEP (S2C Fig, S1 Table).

### mGluR5 and mGluR1 inhibitors protect against prion-mimetic antibodies

**Fig 3:**
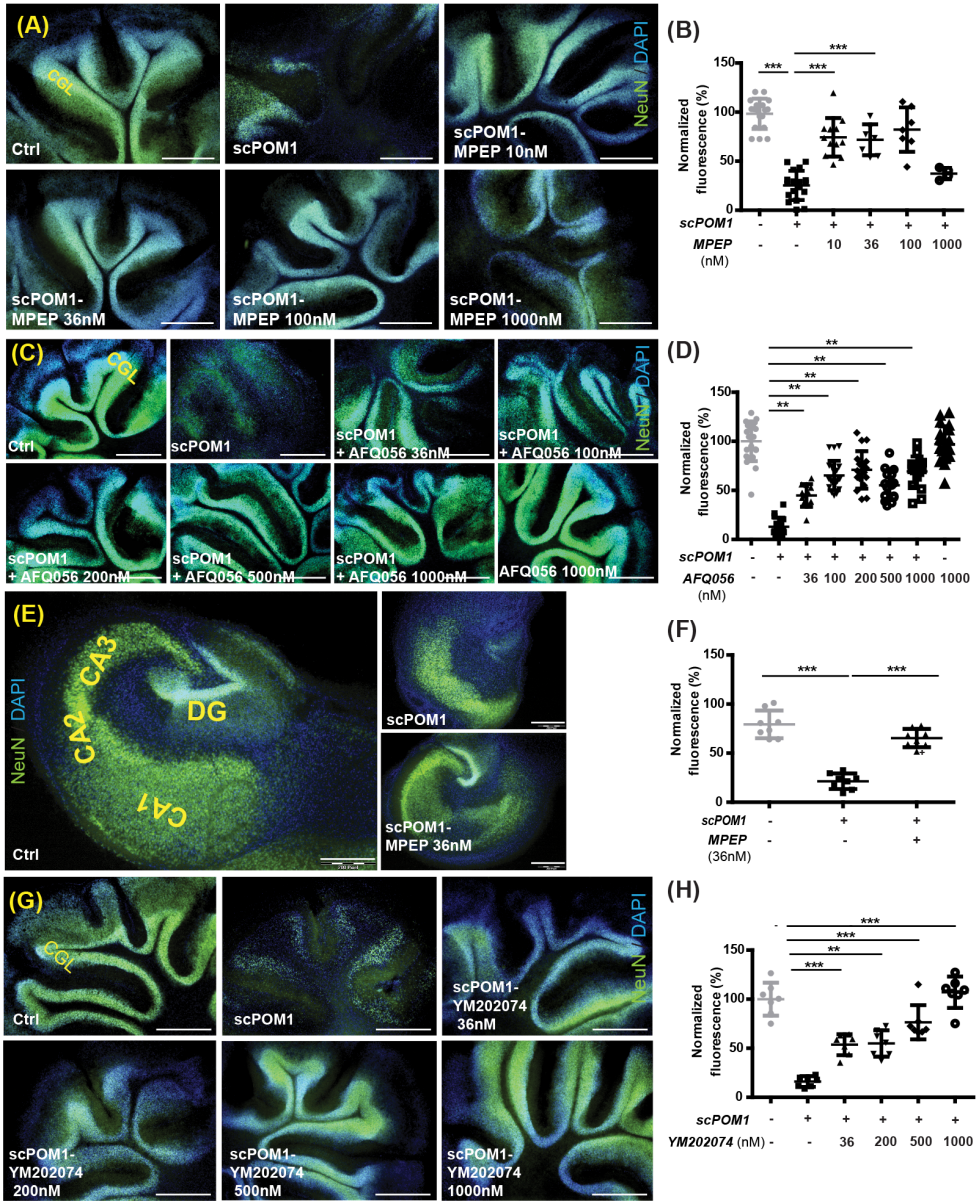
Group-I mGluR inhibition abolishes GDL toxicity in organotypic slice cultures. **(A-B)** Treatment with the mGluR5 inhibitor (MPEP) rescued neurodegeneration in scPOM1-treated COCS from *tg*a*20* mice. **(A)** Ablation of the cerebellar granular layer (CGL) after exposure of *tg*a*20* COCS to scPOM1, and amelioration by MPEP. **(B)** NeuN morphometry of *tg*a*20* slices exposed to scPOM1 or control (scPOM1 blocked with recPrP) and treated with MPEP at 14–28 days post exposure (dpe). **(C)** CGL ablation after exposure to scPOM1, and amelioration by AFQ056. **(D)** NeuN morphometry of *tga20* slices exposed to scPOM1 or control scPOM1 blocked with recPrP and treated with MPEP from 14–22 dpe. **(E-F)** Treatment with MPEP rescued neurodegeneration in *tg*a*20* scPOM1-treated COCS. **(E)** Ablation of the hippocampal neuronal layer induced by exposure of HOCS to scPOM1 (middle), and amelioration by MPEP. **(F)** NeuN morphometry of *tg*a*20* slices exposed to scPOM1 or control (scPOM1 blocked with recPrP) and treated with MPEP from 14–22 dpe. **(G-H)** Treatment with the mGluR1 inhibitor (YM202074) rescued neurodegeneration in *tg*a*20* scPOM1-treated COCS. **(G)** Ablation of the CGL in COCS by exposure to scPOM1, and suppression of toxicity by the mGluR1 antagonist, YM202074. **(H)** NeuN morphometry of *tga20* slices as in panel F, but treated with YM202074 (14–22 dpe). All scale bars: 500μm. For **(B)**, **(D)**, **(F)** and **(H)**: NeuN relative signal intensity as percentage of scPOM1+recPrP control samples. Each dot represents a pool of 7–10 cerebellar slices or 4–6 hippocampal slices cultured in the same well; Data are presented as mean ± s.d.; One-way ANOVA followed by Dunnett’s post-hoc test; **: P < 0.01, ***: P < 0.001.

Antibody-derived molecules targeting the globular domain (GD) of PrP^C^ (termed GDLs) are acutely neurotoxic [40, 41] and activate similar cascades as bona fide prion infection [42]. Single chain POM1 miniantibodies (scPOM1), fusion proteins containing only the variable regions of the heavy (V_H_) and light chains (V_L_) of the antibody connected with a short linker peptide, were previously shown to be sufficient to induce toxicity in COCS [41]. To investigate if pharmacological inhibition of mGluR1 and mGluR5 rescues *GDL* toxicity, we exposed *tg*a*20* COCS to the *GDL* agent scPOM1, followed by YM202074, MPEP and AFQ056 treatments. Treatment with scPOM1 led to almost complete CGL loss within 8 days of treatment. No CGL loss occurred in control treatment where scPOM1 was blocked by pre-incubation with a molar excess of recombinant PrP (recPrP). Treatment with MPEP significantly reduced CGL loss in scPOM1-treated slices. As with prion infections, MPEP treatment (at concentrations as low as 10 nM) was sufficient to rescue the loss of CGL, whereas high concentrations (≥1μM) did not show protective activity (Fig 3A and 3B). Even lower MPEP concentrations (3nM) were sufficient to rescue scPOM1-induced toxicity in COCS (S3E and S3F Fig). AFQ056 and YM202074 treatment (at concentrations as low as 36nM) also significantly reduced the toxicity of scPOM1 (Fig 3C, 3D, 3G and 3H) in COCS.

The protective effect of mGluR1 and mGluR5 inhibitors (YM202074 and MPEP respectively) was further confirmed in wild-type slices. No additional effect was observed upon double MPEP/YM202074 inhibition (S3A and S3B Fig). Similarly to COCS, HOCS treated with scPOM1 exhibited conspicuous toxicity after 8 days of treatment. Neuronal loss was monitored by morphometric analysis of NeuN immunofluorescence, and was readily visible in *GDL*-treated samples, whereas the survival of hippocampal neurons exposed to scPOM1 (Fig 3E and 3F) was greatly increased by treatment with MPEP. In contrast, no protection was observed upon treatment with the selective group III agonist L-2-amino-4-phosphonobutyrate (L-AP4) [43] and the potent group II/III antagonist (*RS*)-α-Cyclopropyl-4-phosphonophenylglycine (CPPG) [44] of metabotropic glutamate receptors (S3C and S3D Fig). Hence toxicity of both infectious prions and prion-mimetic GDLs was prevented by pharmacological inhibition of mGluR1 or mGluR5.

### Toxicity of prions and prion-mimetic antibodies in *Grm5*^*-/-*^ mice

**Fig 4:**
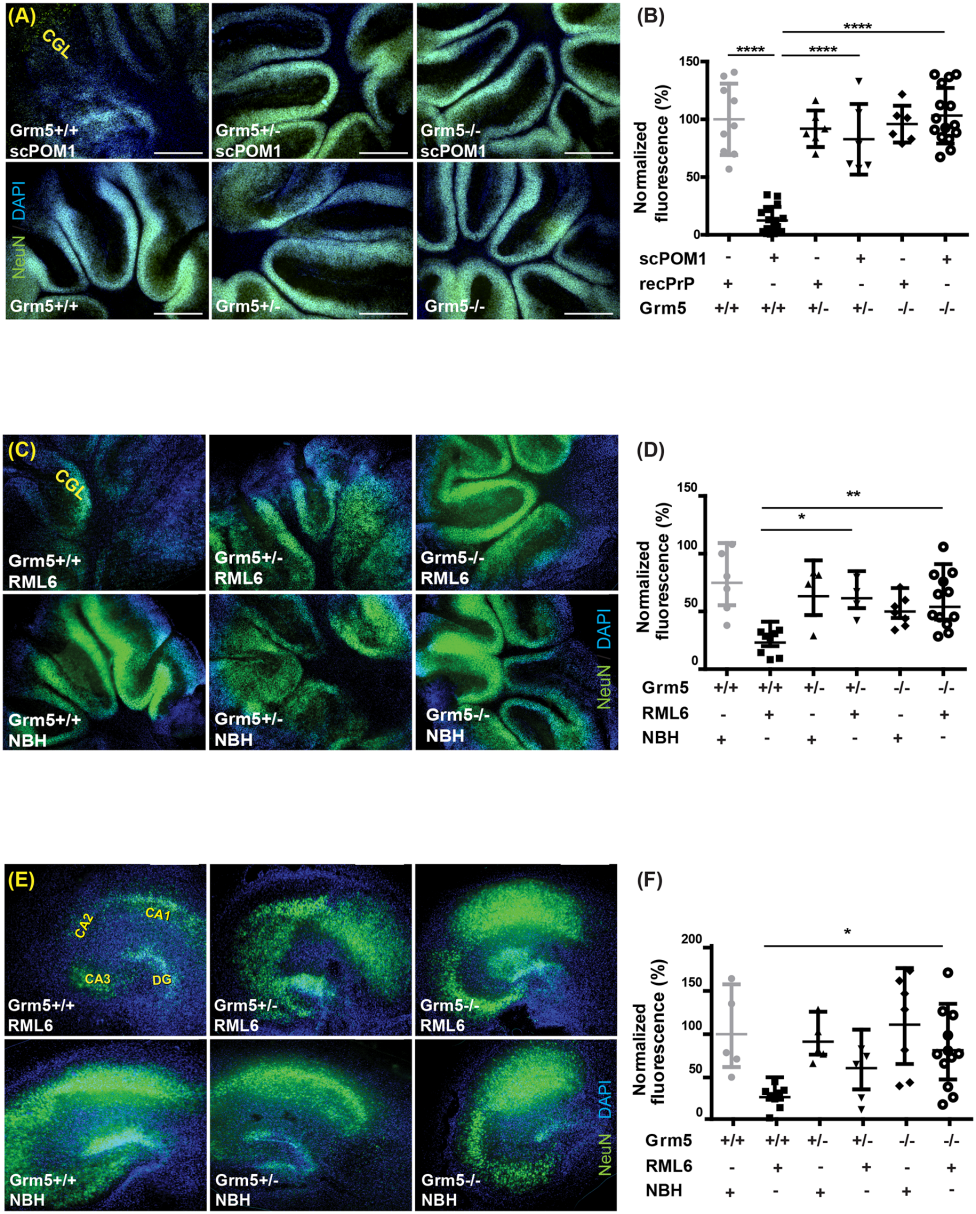
Grm5 ablation protects against GDL and prion-induced neurotoxicity in slice cultures. **(A-B)** scPOM1 induced CGL profound neurotoxicity in *Grm5*^*+/+*^ slices. However, toxicity was much less pronounced in *Grm5*^*+/-*^ and *Grm5*^*-/-*^ slices. **(B)** NeuN morphometry of *Grm5*^*+/-*^ and *Grm5*^*-/-*^ and *Grm5*^*+/+*^ slices exposed to scPOM1 or scPOM1 blocked with recPrP from 14–22 dpe. **(C-D)** CGL ablation induced by RML6 infection in control *Grm5*^*+/+*^ slices, and amelioration in *Grm5*^*+/-*^ and *Grm5*^*-/-*^ slices. Slices were maintained in culture for 60 dpi. **(E-F)** Genetic ablation of *Grm5* rescued prion-induced neurodegeneration in HOCS. **(E)** Representative images of HOCS, showing ablation of the hippocampal neuronal layer induced by RML6 infection in control *Grm5*^*+/+*^ slices, that is significantly ameliorated by the genetic deletion of *Grm5* (*Grm5*^*-/-*^ slices). Slices were maintained in culture for 60 dpi. **(F)** NeuN morphometry of *Grm5*^*+/-*^ and *Grm5*^*-/-*^ and *Grm5*^*+/+*^ slices exposed to RML6 or NBH. RML6-induced neurodegeneration is rescued in the *Grm5*^*+/-*^ and *Grm5*^*-/-*^ HOCS. All scale bars: 500 μm. **(B)**, **(D)** and **(F)**: NeuN relative signal intensity as percentage of control samples (Grm5^+/+^, NBH or POM1+recPrP); each dot corresponds to a pool of 7–10 cerebellar slices or 4–6 hippocampal slices cultured in the same well; Data are presented as mean ± s.d.; One-way ANOVA followed by Dunnett’s post-hoc test. *: P < 0.05, **: P < 0.01, ****: P < 0.0001.

Cerebellar organotypic slice cultures from *Grm5*^-/-^, *Grm5*^+/-^ and *Grm5*^+/+^ littermates were treated with the anti-GD single-chain miniantibody scPOM1 [45], which acts as a prion-mimetic compound. Exposure to scPOM1 led to the loss of cerebellar granular layer (CGL) neurons in *Grm5*^+/+^ slices, but neither in *Grm5*^-/-^ nor in *Grm5*^+/-^ slices (Fig 4A and 4B). We then inoculated cerebellar and hippocampal organotypic slice cultures from *Grm5*^*-/-*^, *Grm5*^*+/-*^ and *Grm5*^+/+^ littermates with RML6 prions or control NBH homogenate. In COCS, both *Grm5*^*-/-*^*and Grm5*^*+/-*^ slices are protected against RML6 toxicity (Fig 4C and 4D). In HOCS, genetic ablation of mGluR5 was protective against prion-induced toxicity (Fig 4E and 4F).

To assess the role of mGluR5 in prion infections *in vivo*, we infected *Grm5*^-/-^, *Grm5*^+/-^ and *Grm5*^+/+^ littermates with RML6 prions (5 log LD_50_). In line with a recently published study [46], no significant difference in survival was observed between *Grm5*^-/-^, *Grm5*^+/-^ and *Grm5*^+/+^ mice (S4A Fig).

The latter finding was unexpected and prompted us to investigate the possibility of compensatory mechanisms. Both group-I metabotropic glutamate receptors, mGluR1 and mGluR5, can associate with PrP^C^ and induce similar intracellular pathways [47] suggesting functional redundancy between these two receptors. In order to detect a possible epistasis between mGluR1 and mGluR5, we assessed mGluR1 and mGluR5 protein levels in cerebellum, cortex and hippocampus of *Grm5*^-/-^, *Grm5*^+/-^ and *Grm5*^+/+^ mice (S4C and S4D Fig).

At 10 days of age, mGluR5 expression was similar in cerebellum, hippocampus and cortex as described [48], whereas mGluR1 was highest in the cerebellum (S4C Fig). Interestingly, we observed an increased expression of mGluR1 in all the three tested regions of *Grm5*^-/-^ brains. We further assessed mGluR1 and mGluR5 levels at later time points (45–180 days). Expression of mGluR5 decreased in all brain regions with increasing age, whereas expression of mGluR1 remained stable. However, we detected increased mGluR1 expression in *Grm5*^-/-^ brains. In the cortex, we observed increased expression of mGluR1 in samples from 45-day old *Grm5*^-/-^ mice compared to *Grm5*^+/+^ littermates (S4D Fig, middle right panel). In the hippocampus, we observed increased expression of mGluR1 in samples from 90-day old *Grm5*^-/-^ mice (S4D Fig, bottom right panel) and in samples from both *Grm5*^+/-^ and *Grm5*^-/-^ 180-day old mice (S4D Fig, lower right panel, lanes 7, 8 & 9 and quantification). In the cerebellum, we observed increased expression of mGluR1 in samples from 90-day old *Grm5*^-/-^ mice compared to wild-type control littermates (S4D Fig, upper right panel).

We then tested whether treatment with MPEP also enhances the expression of mGluR1. mGluR1 expression levels were assessed in whole-brain lysates from 1-year old control wild-type mice, NBH-inoculated wild-type mice, and NBH-inoculated wild-type mice that received MPEP food. However, no differences were observed in the mGluR1 expression levels between the samples (S4B Fig), suggesting that compensatory *Grm1* upregulation is developmentally controlled.

### PrP^C^ interacts with both mGluR1 and mGluR5 in vivo

**Fig 5:**
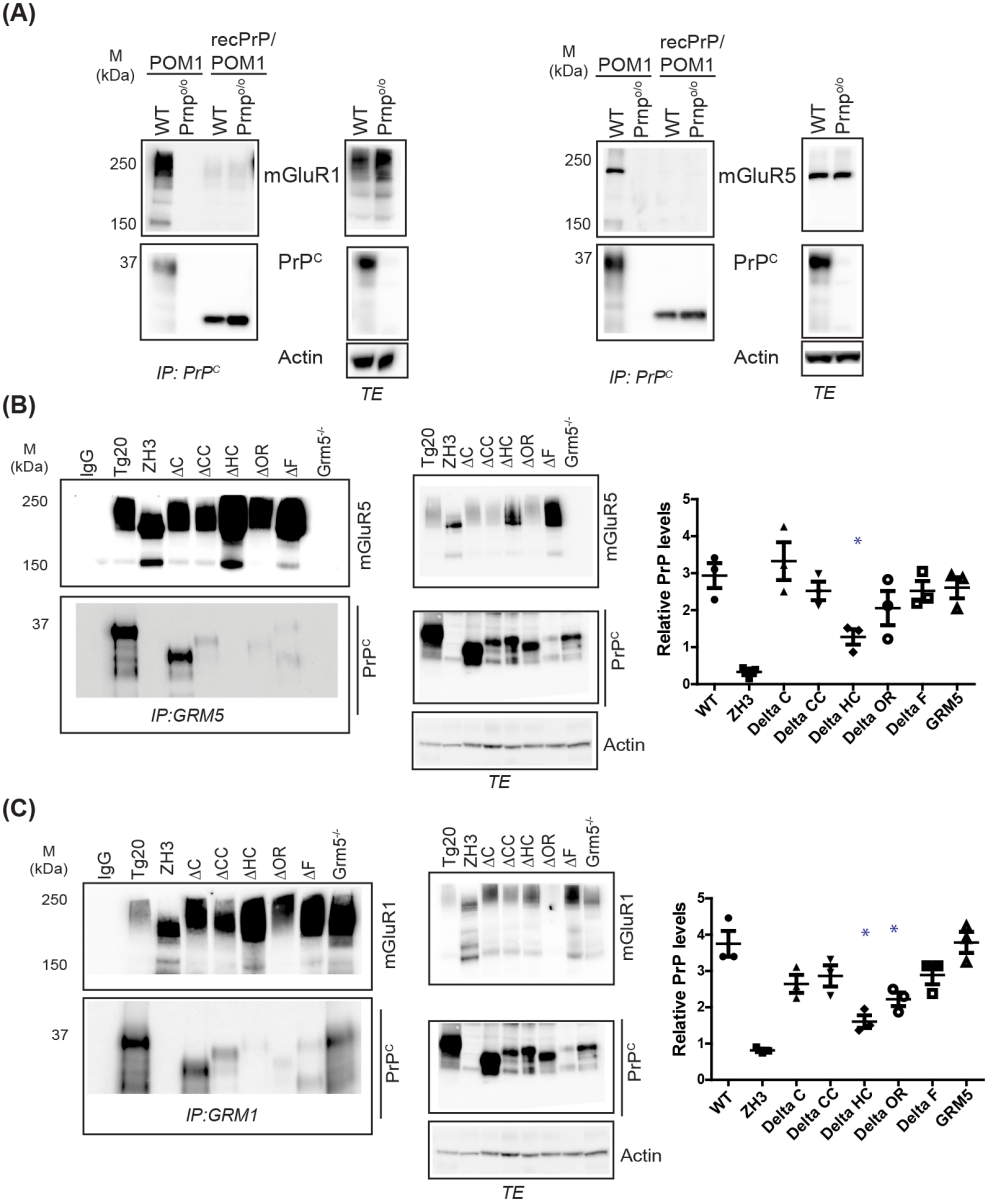
mGluR-interacting domains on PrP^C^. **(A)** Brain homogenate from wild-type (C57BL/6J) and *Prnp*^*o/o*^ mice was subjected to immunoprecipitation by POM1 followed by immunoblotting using polyclonal anti-mGluR5 (right) or anti-mGluR1 (left) and anti-PrP^C^ antibodies. Control conditions (POM1 blocked by recombinant PrP^C^) were run in parallel. The typical mGluR bands of 250kDa and 150kDa were detected in wild-type extract only when immunoblotting with mGluR1 or mGluR5 antibody. Total brain extracts were in parallel subjected to Western blot analysis to control for endogenous levels of mGluR5/1 and PrP^C^. **(B-C)** Mapping the mGluR5 and mGluR1 interacting domains on PrP^C^. Brain homogenate from *Tga20*, *Prnp*^o/o^ (ZH3) and amino proximal deletion mutants of PrP^C^ was subjected to immunoprecipitation by anti-mGluR5 **(B)** or anti-mGluR1 **(C)** antibodies. For detection, we used polyclonal antibodies to mGluR5, mGluR1, and PrP^C^. Deletions encompassing residues 111–134 of PrP^C^ reduced its interaction with mGluR5, whereas deletions of residues 51–90 or 11–134 decreased the interaction with mGluR1. Total brain extracts (TEs) were subjected to Western blot analysis to control for endogenous levels of mGluR5/1 and PrP^C^. Densitometric quantitation of the PrP^C^ signal was normalized over the ratio of PrP/Actin signal in TEs. N = 3–5; One-way ANOVA followed by Tukey’s post-hoc test. Asterisk: P<0.05.

PrP^C^ interacts with mGluR1 and mGluR5 [21, 47]. We confirmed these results by immunoprecipitating brain homogenates from wild-type (C57BL/6J) or *Prnp* knockout mice (*Prnp*^o/o^) using antibody POM1 against PrP^C^, followed by Western blotting with antibodies to mGluR1 and mGluR5. The group-I mGluRs, which migrate as SDS-resistant oligomers at 250kDa [49], were found to co-precipitate with PrP^C^ (Fig 5A). When we blocked the antigen-recognition domain of POM1 with recombinant PrP, mGluR1 and mGluR5 no longer co-precipitated with PrP^C^ (Fig 5A). Western blots of brain lysates (total extracts; TEs) did not reveal any changes in the concentration of mGluR1 and mGluR5 protein between wild-type *tga*20 and *Prnp*^o/o^ homogenates (Figs 5A and S5A). In contrast, mGluR6 and mGluR2/3 did not co-precipitate, confirming the specificity of the interaction (S5B Fig).

The residues 91–153 of PrP^C^ participate to the interaction with mGluR5 [20]. To confirm these findings and to identify the domain of PrP^C^ mediating its interaction with mGluR5, we studied a panel of transgenic mice expressing variants of PrP^C^ bearing deletions in the flexible tail (FT) regions, designated ΔC, ΔCC, ΔF, ΔOR, and ΔHC [50–54] (S5E Fig). In each line of mice, we immunoprecipitated PrP^C^ from brain using POM1 antibody (specific information and binding sites on PrP^C^ are provided in S5F Fig and Table 1) and measured the co-precipitation of mGluR5. Most FT-mutated PrP^C^ variants showed an impaired capacity to co-precipitate mGluR5, with deletions of residues 51–90 and 32–134 showing the most striking reduction (S5C Fig). Conversely, when we performed immunoprecipitations of mGluR5 followed by Western blotting for PrP^C^, we found that deletions spanning residues 111–134 affected the interaction most profoundly (Fig 5B).

**Table 1:** Details of POM antibodies used in the current study.

Antibody	Domain	Epitope	Epitope sequence on PrP^C^
POM1	GD	β1-α1 loop, α1 and α3	138 − 147; 204/208/212
POM2	OR	GQPHGGG/SW	57 − 64, 64 − 72, 72 − 80, 80 − 88
POM3	Hinge	HNQWNK	95 − 100

POM1 binds to the globular domain (GD) of PrP^C^ whereas POM2 binds to a degenerate epitope in the octapeptide repeat region (OR) and POM3 binds at the center of the protein, designated as hinge region. Specific epitopes and the amino acids they span are depicted in the table.

We also analyzed the capacity of PrP^C^ mutants to immunoprecipitate mGluR1. While all examined FT mutations decreased the interaction of PrP^C^ with mGluR1, deletions affecting residues 51–90 showed the most significant reduction (S5D Fig). Immunoprecipitation of mGluR1 revealed that PrP^C^ deletions spanning residues 51–90 and 111–134 had the strongest effect on its interaction with mGluR1 (Fig 5C). Finally, we observed that deletion of mGluR5 had no effect on co-precipitation of PrP^C^ with mGluR1 (Fig 5C), indicating that mGluR1 and mGluR5 interact with PrP^C^ independently of each other.

These results suggest that the interaction domain between PrP^C^ and mGluR5 resides at the N-terminal region of PrP^C^ and is larger than previously inferred, with residues 32–114 participating to the *in vivo* interaction. The interaction domain between PrP^C^ and mGluR1 also resides at the N-terminal region of PrP^C^ and spans residues 51–90 and 111–134.

### MPEP treatment reduces vacuole size and astrogliosis in prion-infected mice

**Fig 6:**
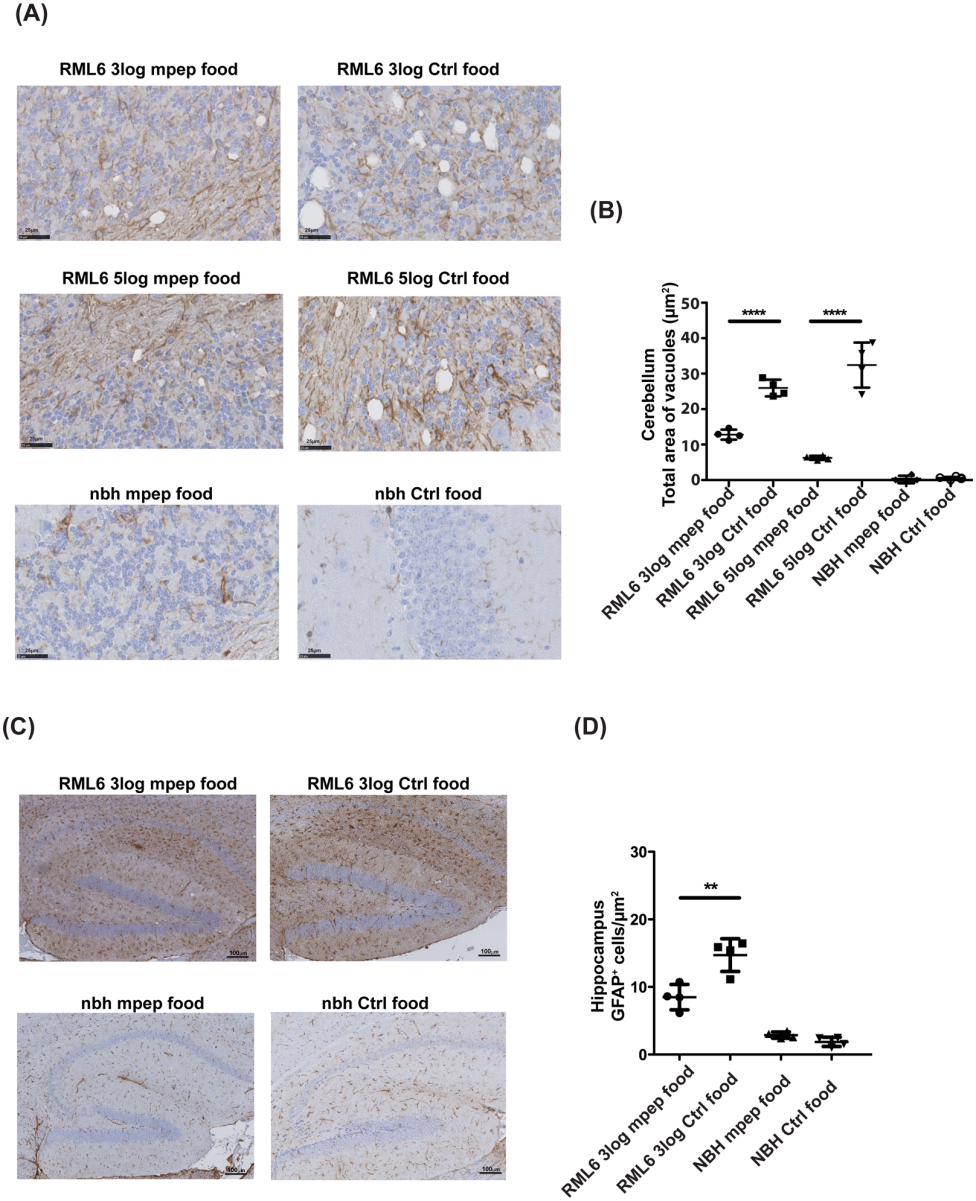
MPEP treatment reduces vacuole size and astrogliosis in prion-infected mice. **(A-B)** GFAP-stained cerebellar sections from C57BL/6J mice injected i.c. with NBH or RML6 prions and treated with control or MPEP-containing food respectively. Image areas as in figure (A) show spongiform vacuoles in the cerebellum. (B) Mean ± SD of vacuole size was quantified as white area over the total area. Each graph shows a treatment group. **(C)** Astrocyte proliferation was analyzed by immunohistochemistry with the GFAP antibody in paraffin-embedded sections of hippocampal areas from C57BL/6J mice injected i.c. with NBH or RML6 prions and treated with control or MPEP-containing food respectively. **(D)** Number of GFAP^+^ cells was quantified in the hippocampus. Each graph corresponds to a treatment group. GFAP staining was markedly reduced in MPEP-treated mice exposed to RML6 (3 log ID_50_ units). Graphs represent mean ± SD GFAP expression, quantified as the percentage of the surface occupied by the GFAP staining over the total measured area. For all graphs, quantification was based on 10 regions of interest per slice, 4 slices per mouse and 4 mice per treatment group. ****P<0.0001, **P<0.01; two-way ANOVA followed by Bonferroni's post-hoc test.

PrP^Sc^ deposition is accompanied by neurodegeneration, vacuole formation and activation of microglia and astrocytes [55]. MPEP treatment did not affect the accumulation of PrP^Sc^ in prion-infected mice and slices (S6A–S6C Fig), yet it reduced vacuole formation. Although the numbers of vacuoles in control and MPEP treated groups were similar, vacuoles were smaller in cerebella of MPEP-treated mice (Fig 6A and 6B). Astrogliosis, assessed by immunohistochemistry for glial fibrillary acidic protein (GFAP), was prominent in terminally sick prion-infected mice but not in NBH-inoculated mice. MPEP treatment reduced the astrogliosis in the hippocampus of prion-infected mice (Fig 6C and 6D), but not in the cerebellar granule cell layer (S6D Fig), as expected from the decreased expression of mGluR5 in the cerebellum of older mice. These findings corroborate the interpretation that MPEP reduces prion toxicity even if it does not affect prion load.

### Prion-mimetic antibodies increase mGluR5 and PrP^C^ translocation to dendritic spines

Clusters of mGluR5 accumulate around excitatory synapses, but are also found at extra-synaptic sites (S7A Fig). Increased size of synaptic mGluR5s clusters is associated with toxic calcium influx [21, 25, 56]. Therefore, we asked whether the prion-mimetic POM1 antibody altered the clustering of mGluR5s. POM2 and POM3 antibodies were also used in parallel (for details about POM antibodies and their epitopes, see Table 1). Specific information and binding sites on PrP^C^ for all antibodies are provided in (S5F Fig, Table 1) and materials and methods.

**Fig 7:**
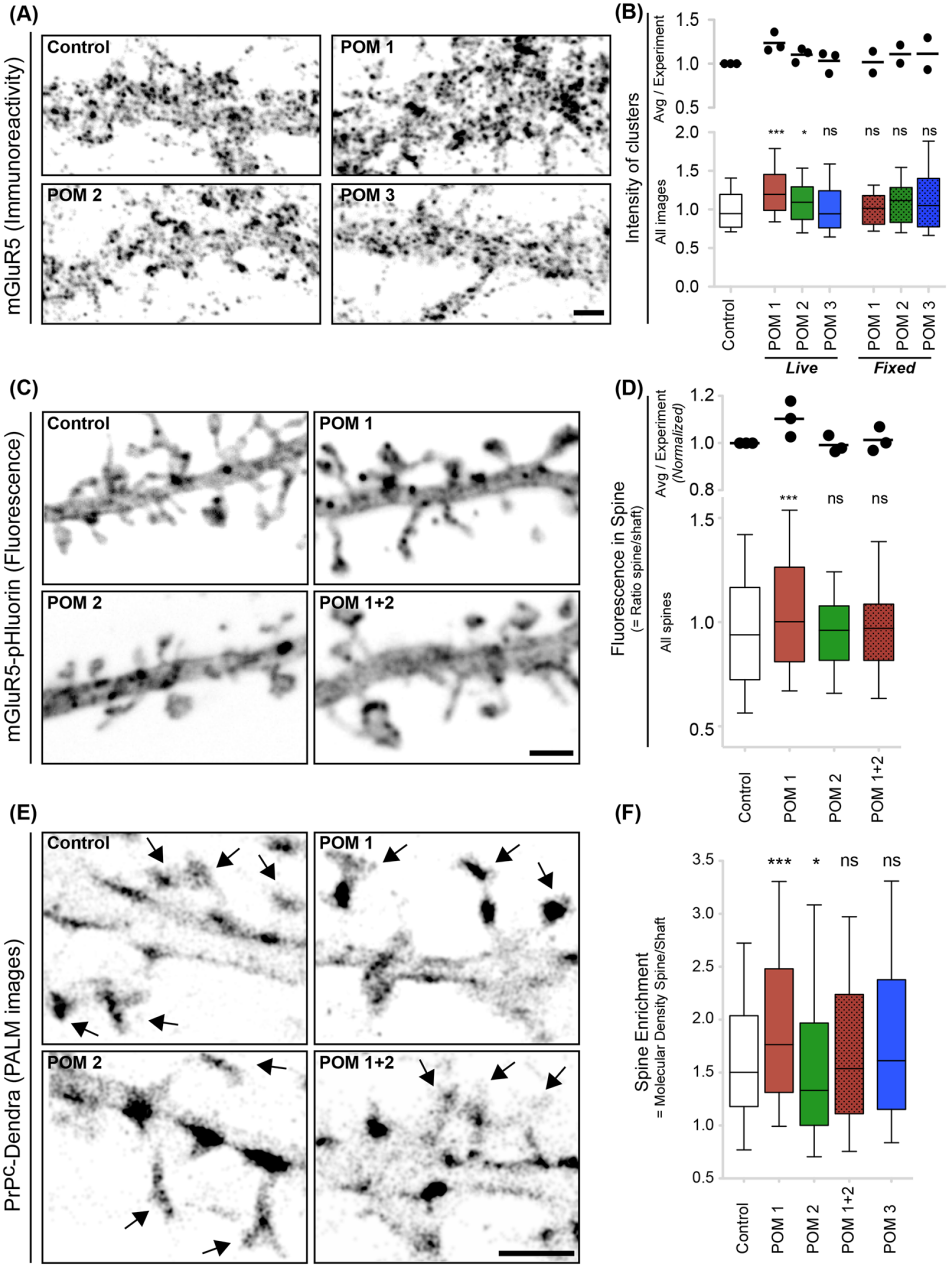
Exposure to Fab_1_-POM1 increases mGluR5 and PrP^C^ translocation to dendritic spines. **(A-B)** mGluR5 immunoreactivity following Fab_1_-POM1 administration to live neurons. Quantification of fluorescence intensity **(B)** showed significantly increased size of mGluR5 clusters following exposure of live neurons to Fab_1_-POM1 compared to Fab_1_-POM2 or Fab_1_-POM3. “*ex vivo*”: antibody administration to live neurons; “*post mortem*”: administration to fixed neurons. The number of images analyzed was: 88 (control), 90 (POM1/live), 59 (POM2/live), 60 (POM3/live), and 30 (POM1/fixed; POM2/fixed; POM3). Results were pooled from three (*ex vivo*) or two (*post-mortem*) independent experiments and distribution of the intensity is plotted (median, quartile, 10–90% distribution). The box plot shows median, quartile and 10–90% distribution and Mann-Whitney test was performed to quantify the differences in distribution. Averaged mGluR5s intensity of clusters per experiment (normalized to control) is also shown in top panel to represent experimental reproducibility (Controls = 1; POM1 (live) = 1.36, 1.19, 1.16; POM2 (live) = 1.17, 1.13, 1.01, POM3 (live) = 1.10, 1.11, 0.89; POM1 (fixed) = 1.14, 0.89; POM2 (fixed) = 1.21, 1.01; POM3 (fixed) = 1.30, 0.93). **(C-D)** Increased mGluR5s immunoreactivity in dendritic spines following Fab_1_-POM1 exposure. **(C)** Representative images showing the expression of mGluR5-pHluorin in untreated and Fab_1_-POM1-treated neurons (1 μg, 1 h). **(D)** Fluorescence ratio (spine/shaft) emphasizing the increase in mGluR5-pHluorin level in spines following exposure to Fab_1_-POM1, but not to Fab_1_-POM2 or a mixture of Fab_1_-POM1 and Fab_1_-POM2. Number of spines analyzed (n): 821 (control), 894 (Fab_1_-POM1), 739 (Fab_1_-POM2), 669 (Fab_1_-POM1+2). The box plot shows median, quartile and 10–90% distribution and Mann-Whitney test was performed. Averaged (normalized to control) spine enrichment value per experiment is also shown (top panel) to represent experimental reproducibility (Controls = 1; POM1 = 1.18, 1.10, 1.03; POM2 = 1.03, 0.96, 0.98; POM1+2 = 1.00, 0.97; 1.07). (**E-F**) Spine enrichment of PrP^C^ following exposure to Fab_1_-POM1. (**E**) Single-molecule detection of PrP^C^-Dendra by photoactivated localization microscopy (PALM) on dendritic spines and shafts for untreated or following antibody treatment (1μg, 1h). (**F**) Ratio of molecular density in spine versus dendritic shaft emphasizing spine-enrichment of PrP^C^-Dendra following exposure to Fab_1_-POM1 but not to other antibodies. Number of spines analyzed (n): 318 (control), 328 (POM1), 364 (POM2), 331 (POM1+2), 416 (POM3). All plots show median, quartile and 10–90% range. Mann-Whitney test; *p<0.05, ***p<0.001, ns = non-significant. Scale bars: 2μm.

Exposure of live neurons to POM1, significantly increased the size of mGluR5s clusters compared to POM2 or POM3 exposure (Fig 7A and 7B), however no change was observed with the NMDA and AMPA receptor clusters (S7B–S7E Fig), suggesting formation of abnormal, potentially deleterious mGluR5 signaling platforms [57]. Next, we examined the fluorescence of dendritic spines of neurons expressing an mGluR5-pHluorin fusion protein. Spines in mGluR5-pHluorin transfected neurons indeed co-localize with post-synaptic marker Homer, which is also a scaffolding protein for mGluR5 (S7F Fig). We observed increased accumulation of mGluR5s in dendritic spines following exposure to POM1, but not to POM2 or POM3 (Fig 7C and 7D).

Both mGluR5 and PrP^C^ are enriched in postsynaptic densities [21]. In order to assess if the changes in mGluR5s level in spines correlated with PrP^C^ level in spines, we performed photo-activated localization microscopy (PALM) on neurons expressing a PrP^C^ tagged with dendra2 fusion protein [58] (Fig 7E). PALM images were obtained from single-molecule detection with a pointing accuracy of 20 nm [58]. The PrP^C^-Dendra fluorescence patterns showed both clustered and diffused staining (Fig 7E, control); we observed an increased enrichment within dendritic spines following POM1 but not POM1+2 exposure (Fig 7E and 7F). Furthermore, exposure to Fab_1_-POM2, which was previously found to protect against POM1 toxicity [41], induced a small but significant reduction in PrP^C^ enrichment within dendritic spines. Therefore, Fab_1_-POM1 and Fab_1_-POM2 may exert opposite effects on the topology and size of mGluR5 clusters, with POM1 inducing abnormal accumulation and translocation to dendritic spines.

## Discussion

Prion toxicity is ultimately mediated by unfolded-protein responses [3, 59], yet it is unclear how these are triggered by PrP^Sc^ which is primarily extracellular. The group-I metabotropic glutamate receptors mGluR5 and mGluR1, G protein-coupled receptors that interact with PrP^C^ [19, 21, 25], may represent one such link. We found that mGluR5 and mGluR1 inhibitors prevented neurodegeneration in prion-infected organotypic slice cultures and protected against prion-mimetic globular-domain ligands [41]. Inhibition of group-I mGluRs may reduce glutamatergic signaling and calcium overload in prion-infected cells [60], similarly to models of Alzheimer’s disease [21, 25].

**Fig 8:**
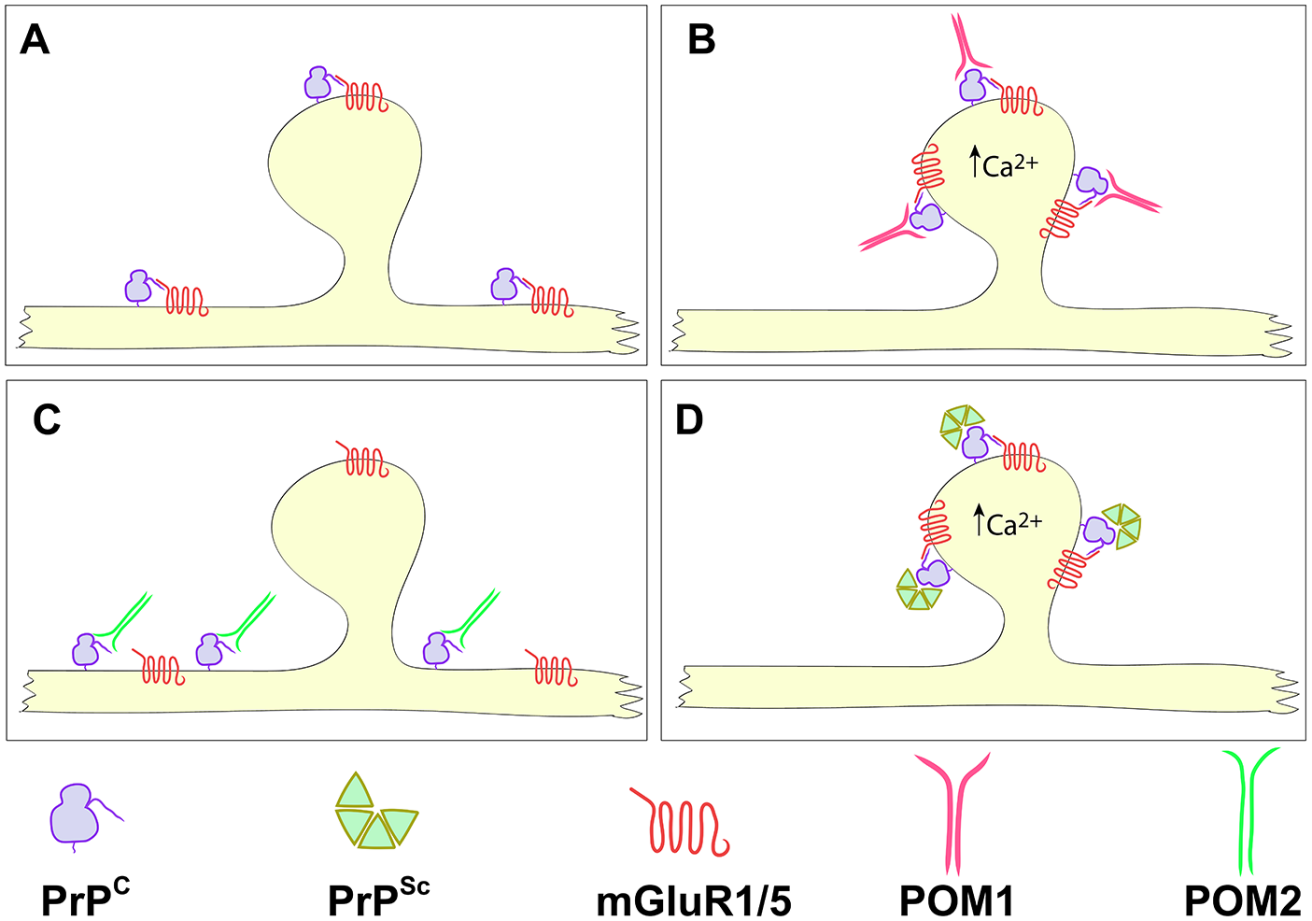
Model of the interactions between mGluR5, PrP^C^, and anti-PrP antibodies. (**A**) In untreated neurons, mGluR5-PrP^C^ complexes are distributed within and outside spines. Upon exposure to prion-mimetic antibodies (**B**), mGluR5 translocates to the spine, where it may enhance neurotoxicity by contributing to a Ca^2+^ overload. (**C**) Exposure to POM2, in contrast, engages the N-terminal “flexible tail” of PrP^C^, thereby making it unavailable to mGluR5. Consequently, mGluR5-PrP^C^ (and possibly also mGluR1-PrP^C^) complexes do not translocate to spines. As a result, POM2 affords functional neuroprotection similarly to mGluR5 antagonists. (**D**) We speculate that prion infection may trigger topological rearrangements similar to those observed after POM1 exposure.

PrP^C^ associates with group-I mGluRs [47] and modulates the signaling activity of mGluR5 [20]. If prion toxicity depends on the direct interaction of PrP^C^ to group-I mGluRs, it may modify the subcellular distribution of mGluR5. Indeed, prion-mimetic antibodies selectively increased clustering of mGluR5 (but not of AMPA and NMDA receptors) in dendritic spine heads, potentially sensitizing them to synaptic glutamate. Prion-mimetic antibodies also increased the level of PrP^C^ in spines, reinforcing the notion that mGluR5 and PrP^C^ are part of the same complex whose accumulation at excitatory synapses instigates neurotoxicity in prion diseases. The impact of POM1 on mGluR5 enrichment within dendritic spines is modest, possibly because only a small fraction of mGluR5 is associated with PrP^C^. Increased cell surface clustering may also slow down endocytosis, thereby increasing the amount of functional mGluR5s [21, 23, 61]. Thus, mGluR5 clustering at synapses may amplify responses to glutamate, thereby exaggerating Ca^2+^ influx and leading to spine loss, a primary event in prion diseases [62]. The POM2 antibody [45] against the Flexible Tail (FT) of PrP^C^ is neuroprotective *in vivo* and *in vitro*. Since both POM2 and mGluR5 bind to the N-terminus of PrP^C^, binding of mGluR5 to PrP^C^ may facilitate its activation whereas POM2 may compete for PrP^C^ binding (Fig 8).

Although mGluR5 inhibition delayed neurological deterioration, survival was only modestly (though significantly) improved. These findings support the concept that mGluR5 inhibition alleviates the symptoms of the disease whereas prion replication progresses unabated. Eventually, the prion load may exert neurotoxicity through mGluR5-independent mechanisms including mGluR1 activation. Not all neurons express mGluR5 [63, 64]; neurons essential for survival may be mGluR5-negative and possibly mGluR1-positive.

Upregulation of mGluR5 can go along with glial activation [56, 65, 66]. We observed reduced GFAP immunoreactivity in hippocampi of MPEP-treated animals (Fig 6C). Conversely, MPEP was unable to suppress glial activation in adult cerebella (S6D Fig) where mGluR5 expression is low, suggesting that dampened neuroinflammation was beneficial.

Genetic ablation of *Grm5* was protective against the toxicity of prion-mimetic antibodies and prion infections in organotypic slices. This effect was haploinsufficient, as hemizygous *Grm5*^+/-^ slices were also protected. Surprisingly, a previous report [46] and this study show that *Grm5* ablation does not ameliorate the clinical manifestation of scrapie *in vivo*. This discrepancy is most likely due to the conspicuous mGluR1 upregulation in *Grm5*^-/-^ and *Grm5*^+/-^ mice.

Co-immunoprecipitations from transgenic mice expressing PrP^C^ with amino-proximal deletions [50–54] showed that both mGluR1 and mGluR5 independently interact with the N-proximal flexible tail of PrP^C^. However, the boundaries of the interacting domain differ, with PrP^C^ residues 32–134 (with residues 51–90 (ΔOR) and 111–134 (ΔHC) acting as important interaction sub-regions) mediating the interaction with mGluR5. The interaction domain appears to extend over the previously reported borders [31]. The interaction domain between PrP^C^ and mGluR1 also resides at the N-terminal region of PrP^C^ and spans residues 51–90 (ΔOR region) and 111–134 (ΔHC region).

Although both *Grm5* genetic deletion and mGluR5 pharmacological inhibition (MPEP) did not prevent prion disease, MPEP significantly improved locomotor abilities until the later stage of disease, decreased the size of spongiform vacuoles, and reduced the extent of hippocampal astrogliosis. These observations are aligned with reports of abnormal expression of group-I mGluRs and mGluR1 signaling in Creutzfeldt-Jakob disease and bovine spongiform encephalopathy [10, 67]. Additional mGluRs may also play a role, and a genome-wide association study identified an mGluR8 variant as a marker for sCJD risk outside the *PRNP* locus [68].

The above data suggest that group-I mGluRs inhibition may attenuate dysfunctions associated with prion diseases, for which there are no disease-modifying therapies. It is unsurprising that mGluR5 antagonists have only a moderate effect on survival, since this therapeutic modality is likely to affect downstream consequences of prion toxicity rather than quenching prion propagation. Because of their orthogonal mode of action, these antagonists may represent ideal compounds for combination therapy with compounds inhibiting prion replication. Because they are well-tolerated and have high bioavailability and blood-brain-barrier penetration [15, 69, 70], mGluR5 antagonists may be useful for enhancing the quality of life of prion patients—a legitimate and important aim even if the overall life expectancy may not be dramatically improved.

## Materials and methods

### Study design

The purpose of this study was to evaluate the therapeutic potential of group I metabotropic glutamate receptor (mGluR1, mGluR5) inhibition in *ex vivo* and *in vivo* models of prion disease. We selected highly specific and well-studied pharmacological inhibitors of mGluR1 and mGluR5, YM202074 and MPEP and AGQ056 respectively, with known specificity and efficiency. To ensure availability of the inhibitors to the brain of prion-infected mice thorough pharmacokinetic and pharmacodynamic analyses were performed. We further extended our study to transgenic mice, knock out for the glutamate receptors being studied. For slice experiments, treatments were randomly assigned to individual wells. For mouse experiments, treatments were randomly assigned to age- and sex-matched mice; experimenters were blinded to experimental group while performing the animal experiments. For experiments with transgenic mice, similar number of heterozygotes and wild-type littermates were included as controls. Mice were sacrificed at the terminal stage of the disease. For analysis, random numbers were assigned to each subject or experimental group.

### Ethics statement

All animal procedures were approved by the local Ethical Committee (Animal Experimentation Committee of the Canton of Zurich, permit 200/2007; 41/2012; 90/2013) in accordance with the Swiss federal, Ethical Principles and Guidelines for Experimenting on animals (3^rd^ edition, 2005). All efforts were made to minimize the suffering and the number of animals used.

### Mice

C57BL/6J wild-type mice were purchased from Jackson laboratories. Male mice were selected because they do not have estrous cycles that can complicate pharmacology. *Prnp*^o/o^ and *Prnp*^o/o^;*tg*a*20*^+/+^ (*tg*a*20*), were on a mixed 129Sv/BL6 background [71, 72]. Transgenic mice expressing mutated PrP^C^ were utilized for immunoprecipitation experiments. The production and relevance to disease phenotype of the Tg mice expressing N-terminal deletion mutants of PrP^C^ (termed ΔC, ΔCC, ΔF, ΔOR, and ΔHC) have been previously reported [50–54]. *Grm5*^*+/-*^ embryos [73, 74] were acquired from Dr. Gasparini and were revitalized at the transgenics facility of the University Hospital of Zurich. *Grm5* null mice were derived from breeding of these mice.

### Pharmacological treatments

2-Methyl-6-(phenylethynyl)-pyridine (MPEP) [36] chronic treatment was initiated at the time of prion inoculation. A dose of 30 mg of MPEP/kg of body weight was selected [75]. The drug was incorporated into chow to achieve voluntary consumption and constant drug administration. Control, untreated groups received the same type of food lacking the drug. For this study, mice between 2 and 4 months of age at the time of prion inoculation/beginning of MPEP treatment were utilized.

To determine PK values in mice fed with food pellets containing MPEP (250mg/kg; Provimi Kliba SA, Rinaustrasse 380, CH-4303 Kaiseraugst), 10 C57BL/6J mice were fed MPEP-food pellets for 15 days and sacrificed to measure the blood/brain ratio of MPEP. Based on an average intake of 3 gram food pellets per day and a body weight of approximately 25 g, a dose of 30mg/kg/day was established. The MPEP concentration was determined by liquid chromatography separation followed by mass spectrometry (LC-MS). Control mice (a total of 8 C57BL/6J mice) received normal food. Mice were sacrificed at two different time points, corresponding to the active and the inactive phase of the mice across the circadian circle and exposures of MPEP in blood and brain were measured.

### Organotypic slice culture preparation

Organotypic cerebellar cultured slices, 350 μm thick, were prepared from 9–12 day-old pups according to a previously published protocol [32]. Organotypic hippocampal cultured slices, 350 μm thick, were prepared from 4–6 day-old pups according to a previously published protocol [33]. Cultures were kept in a standard cell incubator (37°C, 5% CO_2_, 95% humidity) and the culture medium was changed three times per week.

### Prion inoculation and GDLs treatment

Inoculations were performed with either infectious brain lysate (RML6) or non-infectious brain homogenate (NBH). Slices were inoculated (as free-floating sections for 1 h at 4°C) with 100μg brain homogenate per 10 slices. After washing in GBSSK, they were cultured on a 6-well Millicell-CM Biopore PTFE membrane insert (Millipore) according to previously published protocol [60]. Drug-treated *tga20* slices were maintained until 45 dpi, fixed and analyzed by NeuN morphometry (analySIS vc5.0 software). Neurotoxicity was defined as significant NeuN^+^ neuronal layer loss over NBH treatment. Slices prepared from *GRM5*^*-/-*^, *GRM5*^*+/-*^ and *GRM5*^*+/+*^ littermates were maintained until 60 dpi, fixed and analyzed by NeuN morphometry (analySIS vc5.0 software). Neurotoxicity was defined as significant NeuN^+^ neuronal layer loss over NBH treatment.

For globular domain ligand (*GDL*) treatment, toxicity in slices was induced by exposure to ligands, toxic anti- PrP^C^ antibodies targeting the globular domain, such as single chain scPOM1 mini-antibody, after a 14-day recovery period; allowing the initial gliosis induced by tissue preparation to subside, according to previously published protocol [41]. *tg*a*20* COCS were exposed to scPOM1 (200 nM, 8 dpe), or to control treatment (200 nM scPOM1/210nM recPrP, 8 dpe), immunostained for the neuronal marker NeuN and counterstained with DAPI. Slices were imaged and analysed as previously described. Antibody treatment was randomly assigned to individual wells.

### Pharmacological treatment of slices

Treatment with the specific inhibitors 2-Methyl-6-(phenylethynyl)-pyridine (MPEP) [36], AFQ056 (Mavoglurant) [37] or N-cyclohexyl-6-N-methylthiazolo[3,2-a]benzimidazole-2-carboxamide (YM202074) [35] was initiated at the time of GDL addition (14dpe) for the GDL toxicity model (treated slices were maintained until 28 dpe for POM1 treatment and until 22dpe for scPOM1 treatment) [41] and at 21 days post-inoculation (dpi) for prion-infected slices, when PrP^Sc^ accumulation was already discernible [32]. Drug treatments were re-added at every media change [36]. Post-treatment slices were fixed in 4% paraformaldehyde (PFA), immunostained for the neuronal marker NeuN and counterstained with DAPI. Slices were imaged at 4x magnification on a fluorescence microscope (BX-61, Olympus) analyzed by NeuN morphometry (analySIS vc5.0 software). Neuroprotection was defined as significant neuronal layer rescue over toxic-antibody treated, non-drug treated slices.

### Prion inoculations

Inoculum of the RML6 strain of mouse-adapted scrapie prion was prepared from pooled 10% w/v brain homogenates of RML6 terminally sick CD1 mice. C57BL/6J mice were inoculated with serial dilutions (10^−3^ and 10^−5^) of the RML6 inoculum. C57BL/6J mice were injected intracerebrally (i.c.) with 30μl of brain homogenate prepared in a solution of PBS/5% BSA, containing 3log LD_50_ units or 5log LD_50_ units of the RML6 strain. Control mice received 30μl of NBH derived from healthy CD1 mice. Scrapie was diagnosed according to clinical criteria (ataxia, kyphosis, priapism, and hind leg paresis). Mice were sacrificed on the day of onset of terminal clinical signs of scrapie. The operator was blinded to drug treatment.

### Rotarod tests

The rotarod test was used to assess motor coordination and endurance at defined timepoints after prion inoculations. A rotarod machine (Ugo Basile) with five cylinders (3cm diameter) separated by dividers (25cm diameter) in five lanes, each 57mm wide, was utilized. Before the training sessions, the mice were habituated to stay on the rotating rod (4 rpm lowest speed) for 3 sessions lasting 1–2 minutes each and separated by 10 minute intervals. The test phase started 30 minutes after the last habituation session and consisted of 3 trials separated by 15 minute inter-trial intervals. For each test session the mouse was placed on a rotating rod, which accelerated from 5 to 40 rpm. Each test session lasted a maximum of 5min. Latency to fall was assessed when the mouse was no longer capable of riding on the accelerating rod and slipped from the drum. Test sessions were always performed at the same time of the day, mice were tested in a randomized manner and the operator was blind to drug treatment.

### Brain homogenization and immunoprecipitation

Adult *Prnp*^o/o^, *tg*a*20*^+/+^ (*tg*a*20*), and C57BL/6J mice were euthanized and their brains were dissected. Brain samples were snap frozen in liquid nitrogen. Samples were subsequently homogenized in ice cold Lysis Buffer (1% Igepal (NP-40) in 1x PBS, pH 7.4) supplemented with protease (EDTA-free) and phosphatase inhibitor cocktail mix (Roche). Protein concentration was determined using the bicinchoninic acid assay (Pierce). Following immunoprecipitation of PrP^C^ with a specific anti-PrP monoclonal antibody (POM1 or POM2) and addition of Dynabeads M-280 Sheep anti-mouse (#311201D, Thermo Fischer Scientific), samples were prepared in loading buffer (NuPAGE, Invitrogen) and incubated at 37°C for 30 min. For the immunoprecipitation data shown in S4B and S4C Fig, the samples were incubated at 95°C for 5 min; this resulted in disruption of dimers of mGluR5. However this did not have any effect on the immunoprecipitated fractions. The samples were migrated on 4–12% NuPage gels and transfered onto the PVDF membrane. For reverse immunoprecipitation experiments, the subsequent experimental set-up was used. Following immunoprecipitation of mGluR1 or mGluR5 with a specific anti-mGluR1/5 polyclonal antibody (Cell Signalling Technology #12551 or #55920 respectively) and addition of Dynabeads Protein G (#10003D, Thermo Fisher Scientific), samples were prepared in loading buffer (NuPAGE, Invitrogen) and incubated at 37°C for 10–30 min [76]. The samples were migrated on 4–12% NuPage gels and transferred onto the PVDF membrane.

### Antibodies and chemicals

All compounds were purchased from Sigma-Aldrich unless otherwise stated. Monoclonal anti PrP antibody POM1 (1:5000) was generated as described previously [45]. Anti-mGluRs antibodies against representative receptors of each group, targeting the N-terminal domain were utilized: anti-mGluR5 #ab53090 (Abcam) or AB5675 (Millipore), anti-mGluR1 [EPR13540] (ab183712) (Abcam), anti-mGluR2+3 #ab6438 (Abcam) and anti-mGluR6 #AGC-026 (Alomone labs). Secondary antibodies were horseradish peroxidase (HRP)- conjugated rabbit anti–mouse IgG1 (1:10,000, Zymed) and goat anti–rabbit IgG1 (1:10,000, Zymed). Blots were developed using SuperSignal West Pico chemiluminescent substrate (Pierce) and visualized using the VersaDoc system (model 3000, Bio-Rad). Rocky Mountain Laboratory strain (RML; passage #6) prions (RML6) were amplified in CD1 mice by intracerebral inoculation into the lateral forebrain of 30 μl of 1% (wt/vol) brain homogenate. The mGluR5 antagonists MPEP and AFQ056 were kindly provided by Novartis. The mGluR1 antagonist YM202074 was purchased from Tocris Bioscience (Ellisville, USA).

### Immunohistochemistry and NeuN morphometry

Immunohistochemistry of fixed organotypic slices and subsequent NeuN morphometric analysis was performed according to previously published protocols [41, 60].

### Histology and immunohistochemistry

Stainings were performed on sections from brain tissues fixed in formalin and treated with concentrated formic acid to inactivate prions. Partially protease-resistant prion protein deposits, astrogliosis and microglia deposition were visualized by staining brain sections with the SAF84 antibody (1:200, SPI bio), GFAP (1:1000, Millipore) and IBA1 (1:2500, WAKO) respectively on a NexES immunohistochemistry robot (Ventana instruments) using an IVIEW DAB Detection Kit (Ventana), after preceding incubation with protease 1 (Ventana). Images of DAB stained sections were acquired using the NanoZoomer scanner (Hamamatsu Photonics) and NanoZoomer digital pathology software (NDPview; Hamamatsu Photonics). Quantifications of IBA1, GFAP staining and vacuoles in mouse sections were performed on acquired images; regions of interest were drawn on a Digital Image Hub (Leica Biosystems) and analyzed as previously described [77].

### Primary neuronal culture

Hippocampal neurons were prepared from embryonic day 18 (E18) C57/BL6 mice (Janvier Labs, France). Freshly dissociated (trypsin) cells were plated (80,000 cells per 18 mm coverslip per ml) in neuronal attachment media consisting of 10% horse serum, 1 mM sodium pyruvate, and 2 mM glutamine in MEM for 3h. The attachment medium was replaced and cells were maintained in serum-free neurobasal medium supplemented with B27 (1X) and glutamine (2 mM). 300 μl of fresh medium was added once a week.

### Plasmids and transfection

mGluR5-pHluorin construct [78]was generated and kindly provided by Lili Wang and Christian Specht. Dendra2 was inserted between residues Q222 and A223 of mouse prion protein. GluN2A-GFP was kindly provided by Andrea Yao and Pierre Paoletti. Transfection was performed on DIV 17–18 neurons using Lipofectamine as described recently [58]. Transfection medium (TM) was composed of 1 mM sodium pyruvate and 2 mM glutamine in nerobasal medium (Invitrogen). 0.5 μg of plasmid and 2 μl of lipofectamine- 2000 reagent were used for each coverslip. All in vitro experiments were performed on mature neurons (DIV 21–24)

### Immunocytochemistry and image analysis

Immunocytochemistry of mGluR5 (rabbit polyclonal, Millipore, AB5675, 1:200 dilution) or GluR2-AMPA receptor (rabbit polyclonal, Synaptic System, 182103, 1:400 dilution) was performed following methanol fixation / permeabilization (10 min at -20°C; methanol pre-stored at -20°). Image thresholding using wavelet decomposition to identify fluorescent clusters (mGluR5 and GluR2-AMPA immunoreactivity or GluN2-GFP fluorescence) has been described in previous studies [25, 58]. Size of clusters denotes the total fluorescence intensity of the given cluster. Images were acquired using Leica Inverted Spinning Disk microscope (DM5000B, Coolsnap HQ2 camera, Cobolt lasers) using 100X objective (field of view = 1392 x 1040 pixels) and a pixel size of 60.5nm. For estimation of mGluR5 fluorescence within dendritic spines, ratio of fluorescence within a circular region of fixed size (6 pixel) on spine head to the shaft below was measured using ImageJ program.

### Photoactivated localization microscopy (PALM) and analysis

PALM was performed on live neurons expressing PrP^c^-Dendra2 and the microscope setup and lasers used have been recently described in detail [58]. Unconverted Dendra2 has excitation and emission maxima at 490 and 507 nm (green range) while converted Dendra2 protein has excitation and emission maxima at 553 and 573 nm (red range). First, all signal in red channel was photo-bleached to allow detection of single molecule events arising due to the switching of Dendra2 from green to red channel. Single molecule events of Dendra2 were imaged using laser 561 nm (0.5kW, used at 300-400mW) while activating with 405 nm laser (100 mW power, used at 2–5 mW). PrP^c^-Dendra2 was imaged for 5000–6000 frames. Single molecule detections using in-house software has been used and described in previous publications [58]. Density of detections (number/area) of single-molecule on spine head was divided by density of detections over a dendritic shaft to obtain spine enrichment of PrP^C^-Dendra2.

Dendrites were not filled with any additional post-synaptic marker. Mature neurons (DIV 21–24) were transfected with mGluR5-SuperEcliptic pHluorin. The pHluorin-tag allows the visualization of only cell-surface mGluR5s and the neuronal membrane, which is then visually recognizable. We have recently used this plasmid to compute the diffusion dynamics of mGluR5s within dendritic spines [78] In this study, we quantified the spines enrichment of all recognizable spines; considering that visually recognizable spines in mGluR5-pHluorin transfected neurons indeed colocalize with post-synaptic marker, Homer (which is also the scaffold of mGluR5).

### Statistical analysis

Detailed image analysis information is provided in the figure legends. For NeuN morphometric analysis (Figs 1, 3, 4, S1 and S3), NeuN values are normalized to the median NeuN value of the NBH or Ctrl samples respectively. Two-way ANOVA, followed by Bonferroni correction or Log-rank (Mantel-Cox) test was performed in Fig 2, to measure statistical differences between groups. One-way ANOVA followed by Dunnet’s post-hoc test was performed to measure statistical differences between groups. Two-way ANOVA, followed by Bonferroni correction was performed for Fig 4G. For Western Blot quantification in S4 Fig, mGluR1/actin ratios were normalized to the mean Grm5^+/+^ sample mGluR1/actin ratio in each timepoint (45days, 90days, 180days). One-way ANOVA followed by Tukey’s post-hoc test was performed to measure the statistical differences between the groups. For IP quantification in Figs 5 and S5, densitometric quantitation of PrP signal or mGluR1/5 respectively from the immunoprecipitation was normalized over the ration of PrP/Actin or mGluR1/Actin or mGluR5/Actin signal in TEs respectively. One-way ANOVA followed by Tukey’s post-hoc test was performed to measure the statistical differences between the groups. For immunohistochemistry analysis in Figs 6 and S6, number of GFAP^+^ cells or vacuoles was quantified in different brain regions. GFAP expression, quantified as the percentage of the “brown” surface occupied by the GFAP staining over the total measured area. Vacuolation, quantified as the percentage of “white” surface occupied over the total measured area. Two-way ANOVA, followed by Bonferroni correction was performed to measure statistical differences between groups. Non-parametric Mann-Whitney test was performed in Fig 7 to measure the statistical differences between the distributions. GraphPad Prism (GraphPad Software) was chosen for the statistical analysis.

## Supporting information

S1 Fig Treatment with MPEP or YM202074 rescues prion (RML6) toxicity in wild type cerebellar organotypic cultured slices (COCS). Assessment of mGluR5 expression levels in 10-day old samples (COCS and brain homogenates). **(A-B)** Treatment with a mGluR5 or mGluR1 inhibitor (MPEP or YM202074, respectively) rescued neurodegeneration in wild type (C57BL/6J) RML6-treated COCS. **(A)** Fluorescence micrographs of wild type (C57BL/6J) COCS showing degeneration of the cerebellar granular layer (CGL) induced by RML6 infection, that is significantly ameliorated by addition of MPEP or YM202074. **(B)** NeuN morphometry of wild type (C57BL/6J) COCS exposed to RML6 or NBH, and treated with MPEP or YM202074 (dpi: 21–60 days post inoculation). **(C)** Fluorescence micrographs of *tga20* COCS, showing no toxicity on slices treated with high concentrations of MPEP. **(D)** Fluorescent micrographs of *tga20* COCS, infected with RML6 and treated with high concentrations (3-10 μM) of MPEP. High concentrations of MPEP were not protective against prion infection. **(E)** mGluR5 localization in *tga20* COCS imaged by confocal microscopy. The mGluR5 receptor (green) was highly expressed in neuronal and non-neuronal cells in cerebellar slices. Neurons were stained with pAb against NeuN (red); nuclei were counterstained with DAPI (blue). For **(B)** panel: Scatter dot plots represent NeuN relative signal intensity as percentage of NBH samples; each dot corresponds to a pool of 5–8 cerebellar slices cultured in the same well; Data are presented as mean ± s.d.; One-way ANOVA followed by Dunnett’s post-hoc test. For **(A)**, **(C)** and **(D)** panels: Scale bar is 500 μm. For **(E)** panel: Scale bar is 50μm.
(TIF)

S2 Fig MPEP is effectively delivered to the brain, does not induce changes in food and water consumption and rotarod performance of non-infectious brain homogenate (NBH) inoculated mice. (**A**) Control mice injected with NBH and treated with MPEP exhibited stable rotarod performance during the entire test period, up to 23 weeks post-injection. Each dot corresponds to a mouse. Two-way ANOVA per each time point revealed no significant difference in the latency to fall of NBH-injected, MPEP treated mice during the course of the study. (**B**) No significant changes in average food and water consumption were observed between control and treatment (MPEP) groups during the experiment. Experiments were run in parallel. Data are presented as mean ± s.d.; One-way ANOVA followed by Dunnet’s post-hoc test (**C**) Mice treated with control and MPEP food were sacrificed at time points corresponding to the active and the inactive phase across the circadian circle, to determine the exposure of the brain to MPEP. The results indicated the average brain to plasma ratio (Kp) for the MPEP concentration to be around 1; suggesting that the current treatment scheme allows good exposure of the brain to MPEP.
(TIF)

S3 Fig Treatment with MPEP and/or YM202074, but not L-AP4 and CPPG rescues GDL toxicity in wild type cerebellar organotypic cultured slices (COCS). **(A-B)** Treatment with the mGluR5 inhibitor (MPEP) and/ or the mGluR1 inhibitor (YM202074) rescued neurodegeneration in WT (C57BL/6J) scPOM1-treated COCS. **(A)** Fluorescence micrographs of WT COCS showing ablation of the cerebellar granular layer (CGL) induced by scPOM1 treatment, that is ameliorated by addition of MPEP, YM202074 or both inhibitors at low concentrations (C = 100-200nM). **(B)** Graphical representation of NeuN morphometry of *WT* (C57BL/6J) COCS exposed to scPOM1 or control (scPOM1 blocked with recPrP) and treated with MPEP, YM202074, or both. Treatment at 14–22 days post POM1 exposure (dpe). **(C-D)** Treatment with a selective agonist of group III (L-AP4, 500nM) and a potent antagonist of group II-III (CPPG, 200nM) metabotropic glutamate receptors did not rescue neurodegeneration in *tga20* scPOM1-treated COCS. **(D)** NeuN morphometry of *tga20* slices exposed to scPOM1 or control (scPOM1 blocked with recPrP) and treated with L-AP4 or CPPG at 14–22 dpe. **(E)** Fluorescence micrographs of *tga20* COCS showing ablation of the cerebellar granular layer (CGL) induced by scPOM1 and its amelioration by MPEP. **(F)** NeuN morphometry of tga20 COCS exposed to scPOM1 or control (scPOM1 blocked with recPrP) and treated with MPEP at 14–22 dpe. For panels **(B)**, **(D)** and **(F)**: Scatter dot plots represent NeuN relative signal intensity as percentage of scPOM1+recPrP control samples; each dot corresponds to a pool of 7–10 cerebellar slices in the same well; Data are presented as mean ± s.d.; One-way ANOVA followed by Dunnett’s post-hoc test; ***: P < 0.001. For **(A)**, **(C)** and **(E)** panels: Scale bar is 500μm.
(TIF)

S4 Fig Grm5 deletion induces compensatory mGluR1 upregulation and does not prolong survival of prion-infected mice. **(A)** Survival of *Grm5*^*+/+*^, *Grm5*^*+/-*^ and *Grm5*^*-/-*^ mice inoculated i.c. with 5 log LD_50_ units of RML6, n = 4–6 males per group. Each dot corresponds to a mouse. Two-way ANOVA per each time point revealed a non-significant difference between *Grm5*^*+/+*^, *Grm5*^*+/-*^ and *Grm5*^*-/-*^ groups. **(B)** Total brain extracts from mice inoculated with NBH and received control or MPEP food, as well as control WT brain lysates, were subjected to western blot analysis to evaluate whether MPEP treatment changes the expression of mGluR1 receptor. No differences were observed in the mGluR1 expression levels between the samples. **(C)** Cerebellar extracts from *Grm5*^-/-^, *Grm5*^+/-^ and *Grm5*^+/+^ mice, collected at postnatal day 10 (comparable with the organotypic slices), were subjected to western blot analysis to control for endogenous levels of mGluR5. mGluR5 expression in the cerebellum was similar to that of hippocampus and cortex. **(D)** Epistatic interactions between mGluR1 and mGluR5 receptors. Brain extracts from cerebellum, cortex and hippocampus of 45, 90 and 180-day old *Grm5*^*-/-*^, *Grm5*^*+/-*^ and *Grm5*^*+/+*^ mice were subjected to western blot analysis for mGluR1 and mGluR5. With increasing age mGluR5 expression decreased in all brain regions. Expression of mGluR1 remained stable in all genotypes. However, increased mGluR1 expression was detected in samples from *Grm5*^*-/-*^ mice. In hippocampi, we observed higher expression of mGluR1 in samples from *Grm5*^*-/-*^ mice at 90 and 180 days of age than in heterozygous and wild-type littermates (bottom right panel). In the cortex, increased expression of mGluR1 in samples from *Grm5*^*-/-*^ mice were observed at the earliest timepoint (45 days). In cerebellum, we observed increased expression of mGluR1 in *Grm5*^*-/-*^ mice at the intermediate timepoint (90 day). Expression levels of mGluR1 were similar in *Grm5*^*-/-*^ and *Grm5*^*+/+*^ samples at all ages except in 180-day old hippocampal samples (lower panel, lanes 7 and 8). Graph bars represent normalized mGluR1 signal; N = 3–5; One-way ANOVA followed by Tukey’s post-hoc test; n*: P<0.05.
(TIF)

S5 Fig PrP^C^ specifically interacts with mGluR1/5 and not with group II and III mGluRs. **(A)** Total brain extacts from wild-type (C57BL/6J), *Tga*20 and *Prnp*^o/o^ mice was subjected to western blot analysis for the endogenous levels of mGluR5 and mGluR1. Expression of mGlur5/1 was similar in all the three mice model systems. **(B)** Brain homogenate from wild-type (C57BL/6J) and *Prnp*^o/o^ mice was subjected to immunoprecipitation by POM1 followed by immunoblotting using polyclonal anti-mGluR2/3 and anti-mGluR6, or anti-PrP^C^ antibodies. mGluR2/3 and mGluR6 did not coprecipitate with PrP^C^. Total brain extracts were in parallel subjected to Western blot analysis to control for endogenous levels of mGluR2/3 or 6 and PrP^C^. **(C-D)** Mapping the mGluR5 and mGluR1 interacting domains on PrP^C^. Brain homogenate from wild-type, *Prnp*^o/o^ (ZH3) and amino proximal deletion mutants of PrP^C^ was subjected to immunoprecipitation by POM1, followed by immunoblotting using polyclonal anti-mGluR5 **(C)** or anti-mGluR1 **(D)** and anti-PrP^C^ antibodies. Deletions extending from residues 51–90 and 32–134, corresponding to the OR (octapeptide repeat region) and the flexible tail of PrP^C^, reduced the interaction with mGluR5, whereas deletions extending from residues 51 to 90, corresponding to the OR region of PrP^C^, decreased the interaction with mGluR1. Total brain extracts (TEs) were subjected to Western blot analysis to control for endogenous levels of mGluR5/1 and PrP^C^. Densitometric quantitation of mGluR1 or mGluR5 signal from the immunoprecipitation was normalized over the ration of Grm/Actin signal in TEs. Graphs represents mGluR1 or mGluR5 relative signal intensity; N = 3–5; One-way ANOVA followed by Tukey’s post-hoc test; n*: P<0.05. **: band corresponding to recombinant PrP. **(E)** Schematic representation of PrP^C^ deletion mutant**s**. Toxic POM1 antibody binds to a1-a3 helixes (residues 138–147; 204/208/212), innocuous POM2 antibody binds to octapeptide repeat (OR) region (residues 57–88), whereas POM3 antibody binds to residues 95–100 on PrP^C^.
(TIF)

S6 Fig PrP^Sc^ accumulation in prion-infected slices or in the brain of prion-infected mice is not altered by MPEP treatment. **(A)** Total PrP and PrP^Sc^ levels (detected by addition of proteinase K (PK)) in homogenates from different brain regions (hippocampus and cerebellum) of terminal C57BL/6J mice injected i.c. with NBH or RML6 prions and treated with control or MPEP-containing food respectively. Control NBH and RML6 samples, with or without addition of PK were run in parallel. **(B)** Representative images of SAF84-stained cerebellar and hippocampal sections from C57BL/6J mice injected i.c. with NBH or RML6 prions and treated with control or MPEP-containing food respectively. The levels of PrP^Sc^ (detected by SAF84 immunohistochemistry) are similar in brain sections from prion-infected mice treated with control or MPEP-containing food. **(C)** Total PrP and PrP^Sc^ levels (detected by addition of proteinase K (PK)) in homogenates from RML6 infected cerebellar slices prepared from *tga20* or PrP^o/o^ mice. Cerebellar slices infected with RML6 prions were also treated with MPEP according to the previously described protocol. Control NBH samples, with or without addition of PK were run in parallel. **(D)** Astrocyte proliferation was analyzed by immunohistochemistry with the GFAP antibody on cerebellar sections from *C57BL/6J* mice injected i.c. with NBH or RML6 prions and treated with control or MPEP-containing food respectively. Number of GFAP^+^ cells was quantified in the cerebellar granular layer (CGL). Dot blots represent mean ± SD GFAP expression, quantified as the percentage of the surface occupied by the GFAP staining over the total measured area; 10 regions of interest per slice, 4 slices per mouse and 4 mice per treatment group were used for quantification; two-way ANOVA followed by Bonferroni's post-hoc test.
(TIF)

S7 Fig POM antibodies do not alter AMPA and NMDA receptor clustering. **(A)** Immunoreactivity of mGluR5s and PSD95 in cultured hippocampal neurons. Threshold images show the identified clusters. Arrow indicates that synaptic clusters co-localize with mGluR5 clusters. **(B)** Representative image (control condition) showing the immunoreactivity of GluR2 subunit of AMPA receptor following methanol fixation / permeabilization. Scale bar: 2 μm. **(C)** Quantification of the fluorescence intensity indicate that cluster size was not modified following POM antibodies application (One-way ANOVA with Dunnett’s post-hoc test relative to control; field of view (n): Control-22, POM1-22, POM2-22, POM3-22 from 2-independent experiments). **(D)** Representative image (control condition) showing the fluorescence of GluN2A-GFP subunit of NMDA receptor ~48 h after transfection and paraformaldehyde fixation. Scale bar: 2μm. **(E)** Quantification of fluorescence intensity indicate that the cluster size was not modified following POM antibodies application (One-way ANOVA with Dunnett’s post-hoc test relative to control; field of view (n): Control-22, POM1-22, POM2-22, POM3-20 from 2-independent experiments). **(F)** Representative images showing that the spines in mGluR5-SEP transfected neurons co-localize with the post-synaptic marker, Homer (which is also the scaffold of mGluR5s).
(TIF)

S1 Table MPEP values (diurnal measurements) in brain and blood samples. MPEP levels were assessed in blood and brain of mice at two circadian points within a day (light/dark cycle). The brain-to-plasma ratios were calculated based on this analysis and is represented in the tables. The upper table contains the levels of MPEP in brain and the lower table contains the levels of MPEP in blood.
(PDF)
