# Supplementary figures and images for: Inhibition of group-I metabotropic glutamate receptors protects against prion toxicity
Source: PLoS Pathog. 2017 Nov 27;13(11):e1006733. doi: 10.1371/journal.ppat.1006733 (PMC5720820; doi:10.1371/journal.ppat.1006733)

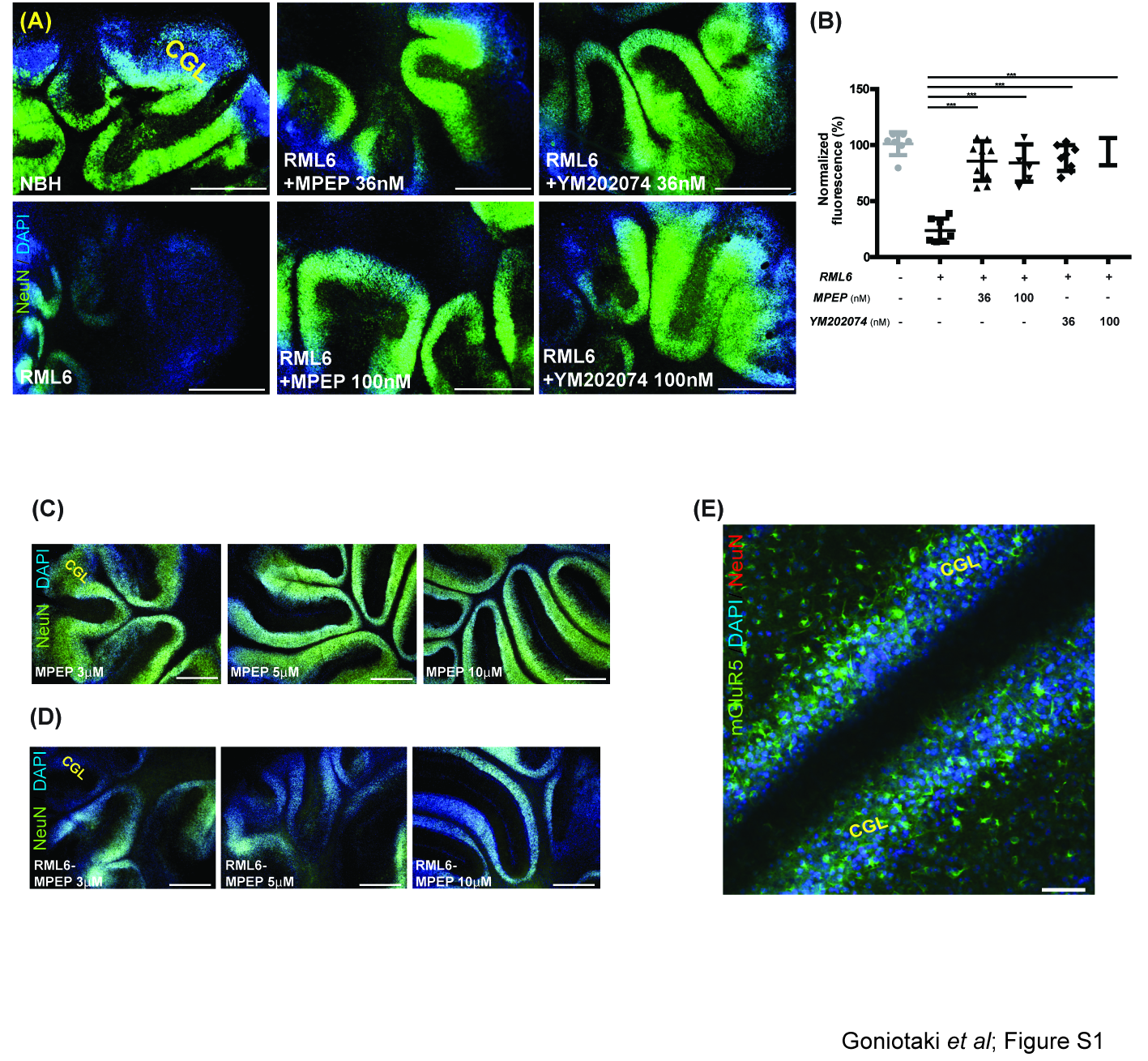

Supplement: S1 Fig — (A-B) Treatment with a mGluR5 or mGluR1 inhibitor (MPEP or YM202074, respectively) rescued neurodegeneration in wild type (C57BL/6J) RML6-treated COCS. (A) Fluorescence micrographs of wild type (C57BL/6J) COCS showing degeneration of the cerebellar granular layer (CGL) induced by RML6 infection, that is significantly ameliorated by addition of MPEP or YM202074. (B) NeuN morphometry of wild type (C57BL/6J) COCS exposed to RML6 or NBH, and treated with MPEP or YM202074 (dpi: 21–60 days post inoculation). (C) Fluorescence micrographs of tga20 COCS, showing no toxicity on slices treated with high concentrations of MPEP. (D) Fluorescent micrographs of tga20 COCS, infected with RML6 and treated with high concentrations (3-10 μM) of MPEP. High concentrations of MPEP were not protective against prion infection. (E) mGluR5 localization in tga20 COCS imaged by confocal microscopy. The mGluR5 receptor (green) was highly expressed in neuronal and non-neuronal cells in cerebellar slices. Neurons were stained with pAb against NeuN (red); nuclei were counterstained with DAPI (blue). For (B) panel: Scatter dot plots represent NeuN relative signal intensity as percentage of NBH samples; each dot corresponds to a pool of 5–8 cerebellar slices cultured in the same well; Data are presented as mean ± s.d.; One-way ANOVA followed by Dunnett’s post-hoc test. For (A), (C) and (D) panels: Scale bar is 500 μm. For (E) panel: Scale bar is 50μm. (TIF) [file ppat.1006733.s001.tif]

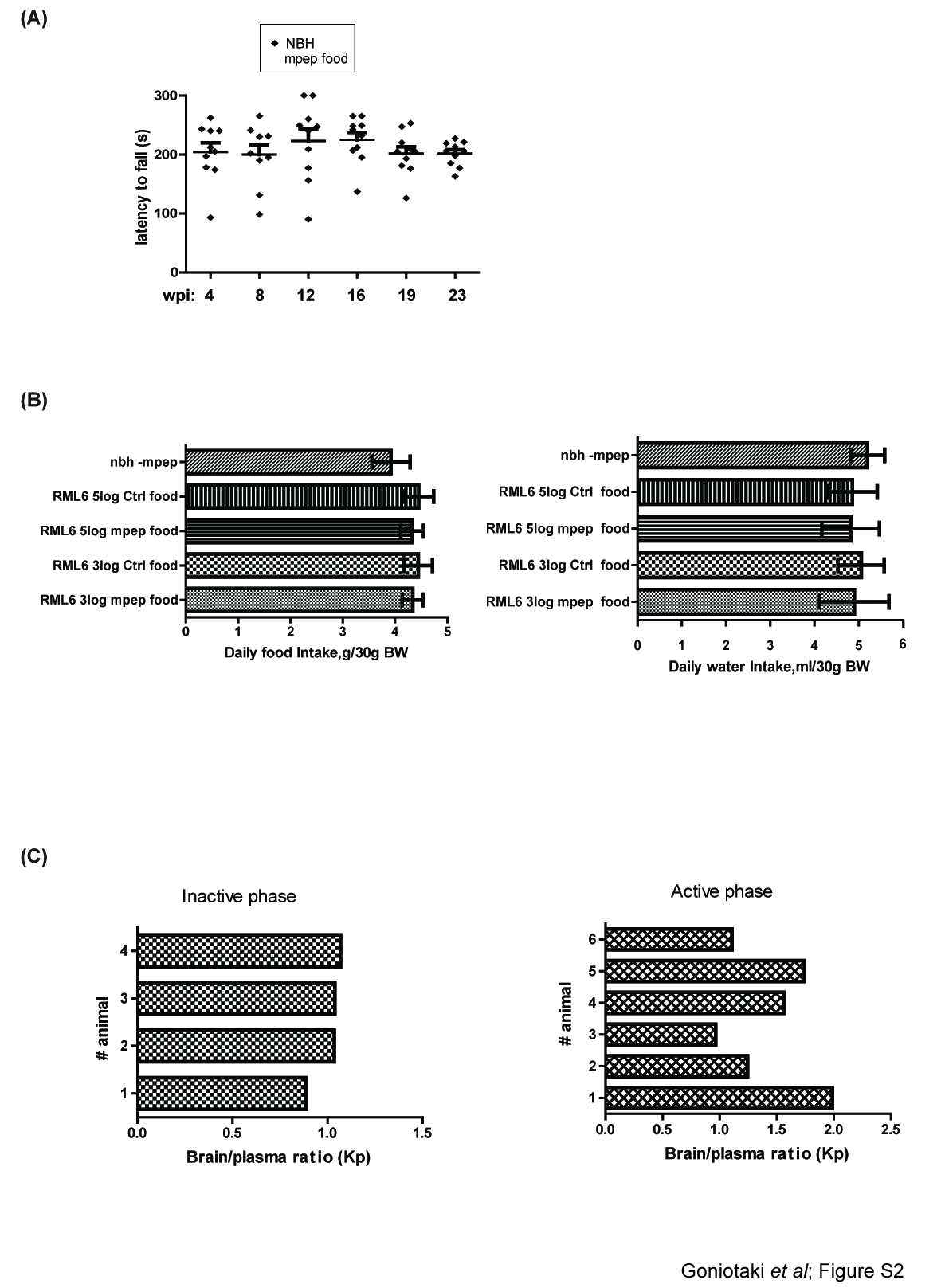

Supplement: S2 Fig — (A) Control mice injected with NBH and treated with MPEP exhibited stable rotarod performance during the entire test period, up to 23 weeks post-injection. Each dot corresponds to a mouse. Two-way ANOVA per each time point revealed no significant difference in the latency to fall of NBH-injected, MPEP treated mice during the course of the study. (B) No significant changes in average food and water consumption were observed between control and treatment (MPEP) groups during the experiment. Experiments were run in parallel. Data are presented as mean ± s.d.; One-way ANOVA followed by Dunnet’s post-hoc test (C) Mice treated with control and MPEP food were sacrificed at time points corresponding to the active and the inactive phase across the circadian circle, to determine the exposure of the brain to MPEP. The results indicated the average brain to plasma ratio (Kp) for the MPEP concentration to be around 1; suggesting that the current treatment scheme allows good exposure of the brain to MPEP. (TIF) [file ppat.1006733.s002.tif]

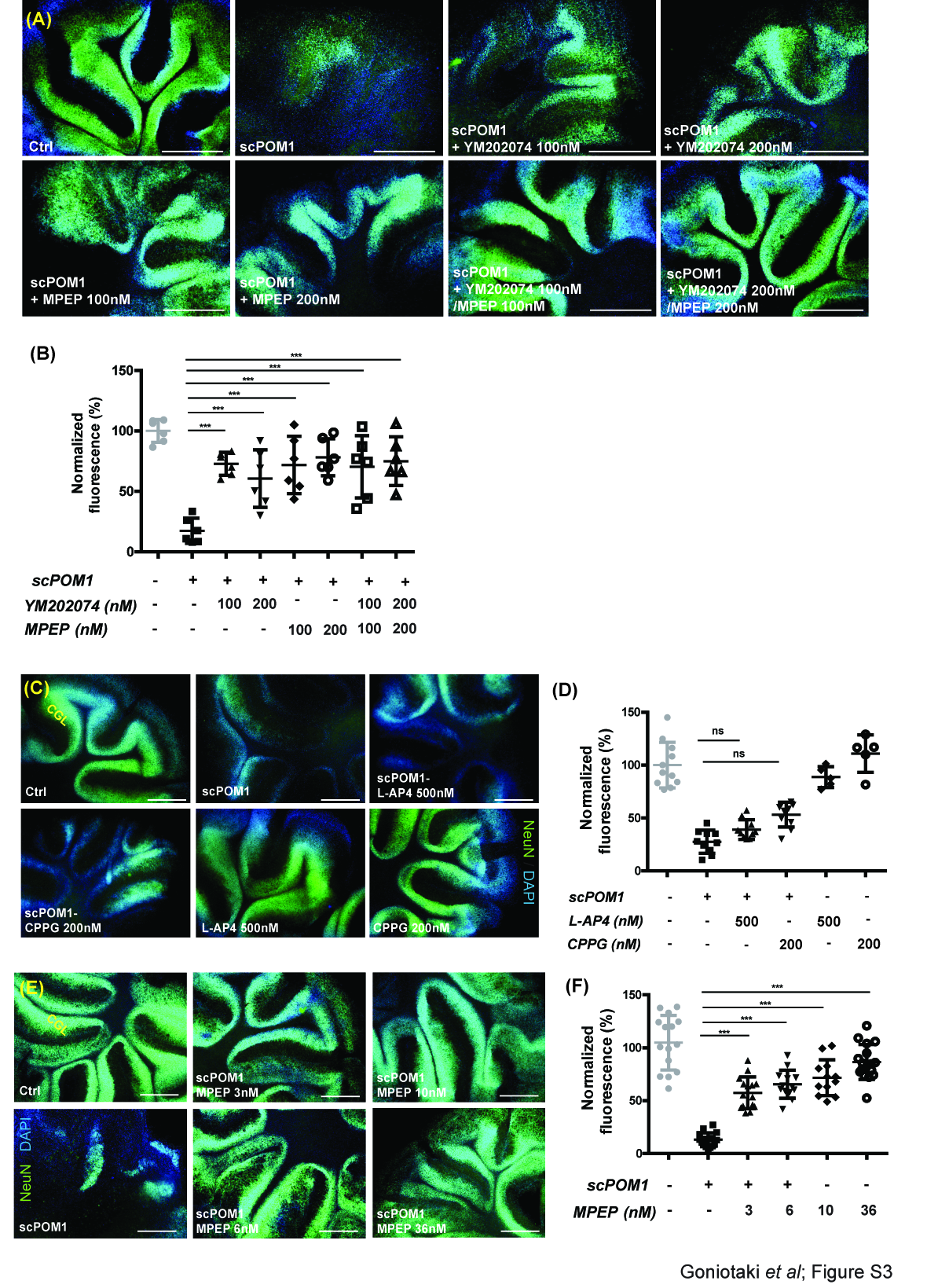

Supplement: S3 Fig — (A-B) Treatment with the mGluR5 inhibitor (MPEP) and/ or the mGluR1 inhibitor (YM202074) rescued neurodegeneration in WT (C57BL/6J) scPOM1-treated COCS. (A) Fluorescence micrographs of WT COCS showing ablation of the cerebellar granular layer (CGL) induced by scPOM1 treatment, that is ameliorated by addition of MPEP, YM202074 or both inhibitors at low concentrations (C = 100-200nM). (B) Graphical representation of NeuN morphometry of WT (C57BL/6J) COCS exposed to scPOM1 or control (scPOM1 blocked with recPrP) and treated with MPEP, YM202074, or both. Treatment at 14–22 days post POM1 exposure (dpe). (C-D) Treatment with a selective agonist of group III (L-AP4, 500nM) and a potent antagonist of group II-III (CPPG, 200nM) metabotropic glutamate receptors did not rescue neurodegeneration in tga20 scPOM1-treated COCS. (D) NeuN morphometry of tga20 slices exposed to scPOM1 or control (scPOM1 blocked with recPrP) and treated with L-AP4 or CPPG at 14–22 dpe. (E) Fluorescence micrographs of tga20 COCS showing ablation of the cerebellar granular layer (CGL) induced by scPOM1 and its amelioration by MPEP. (F) NeuN morphometry of tga20 COCS exposed to scPOM1 or control (scPOM1 blocked with recPrP) and treated with MPEP at 14–22 dpe. For panels (B), (D) and (F): Scatter dot plots represent NeuN relative signal intensity as percentage of scPOM1+recPrP control samples; each dot corresponds to a pool of 7–10 cerebellar slices in the same well; Data are presented as mean ± s.d.; One-way ANOVA followed by Dunnett’s post-hoc test; ***: P < 0.001. For (A), (C) and (E) panels: Scale bar is 500μm. (TIF) [file ppat.1006733.s003.tif]

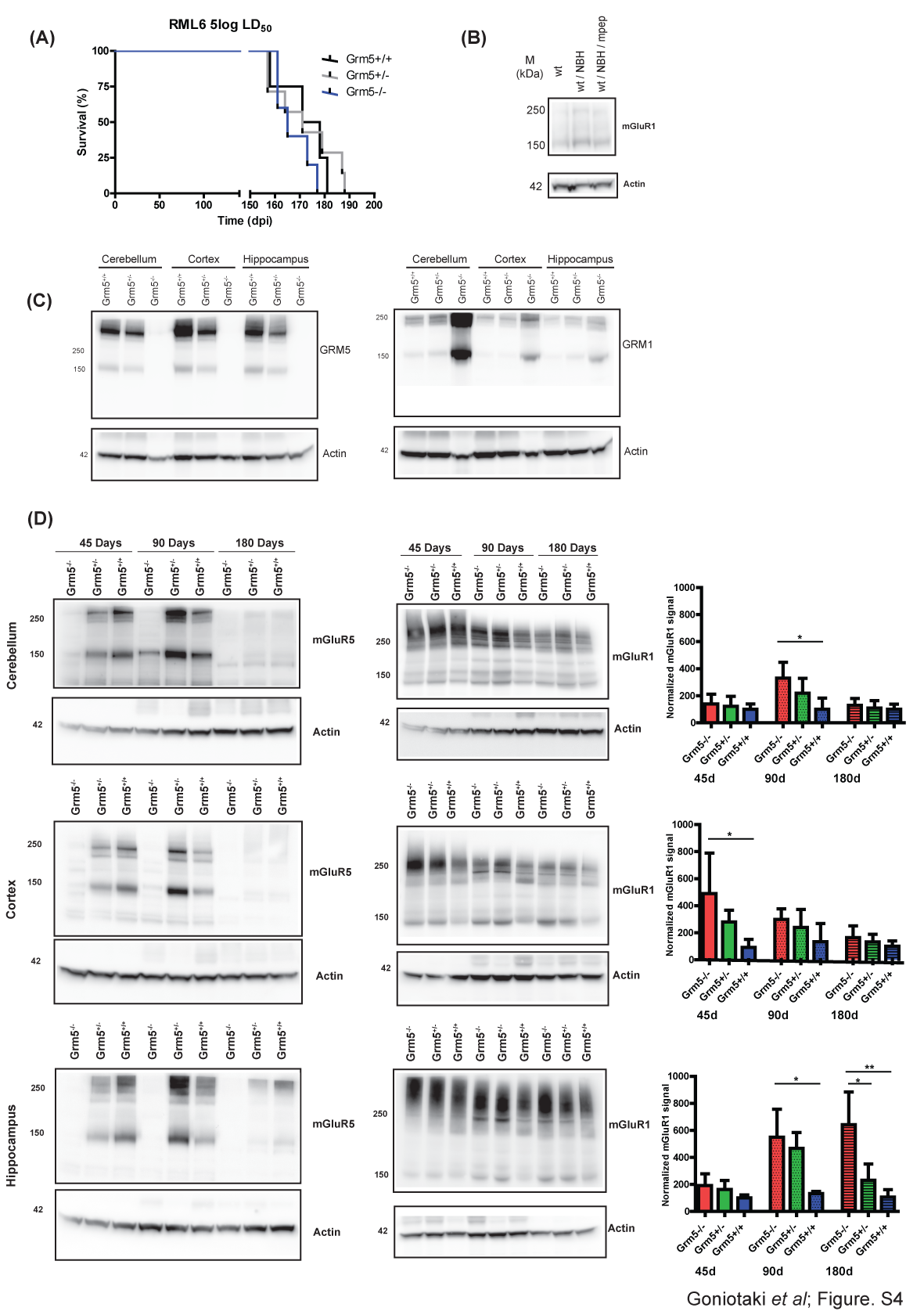

Supplement: S4 Fig — (A) Survival of Grm5+/+, Grm5+/- and Grm5-/- mice inoculated i.c. with 5 log LD50 units of RML6, n = 4–6 males per group. Each dot corresponds to a mouse. Two-way ANOVA per each time point revealed a non-significant difference between Grm5+/+, Grm5+/- and Grm5-/- groups. (B) Total brain extracts from mice inoculated with NBH and received control or MPEP food, as well as control WT brain lysates, were subjected to western blot analysis to evaluate whether MPEP treatment changes the expression of mGluR1 receptor. No differences were observed in the mGluR1 expression levels between the samples. (C) Cerebellar extracts from Grm5-/-, Grm5+/- and Grm5+/+ mice, collected at postnatal day 10 (comparable with the organotypic slices), were subjected to western blot analysis to control for endogenous levels of mGluR5. mGluR5 expression in the cerebellum was similar to that of hippocampus and cortex. (D) Epistatic interactions between mGluR1 and mGluR5 receptors. Brain extracts from cerebellum, cortex and hippocampus of 45, 90 and 180-day old Grm5-/-, Grm5+/- and Grm5+/+ mice were subjected to western blot analysis for mGluR1 and mGluR5. With increasing age mGluR5 expression decreased in all brain regions. Expression of mGluR1 remained stable in all genotypes. However, increased mGluR1 expression was detected in samples from Grm5-/- mice. In hippocampi, we observed higher expression of mGluR1 in samples from Grm5-/- mice at 90 and 180 days of age than in heterozygous and wild-type littermates (bottom right panel). In the cortex, increased expression of mGluR1 in samples from Grm5-/- mice were observed at the earliest timepoint (45 days). In cerebellum, we observed increased expression of mGluR1 in Grm5-/- mice at the intermediate timepoint (90 day). Expression levels of mGluR1 were similar in Grm5-/- and Grm5+/+ samples at all ages except in 180-day old hippocampal samples (lower panel, lanes 7 and 8). Graph bars represent normalized mGluR1 signal; N = 3–5; One-way ANOVA follow [file ppat.1006733.s004.tif]

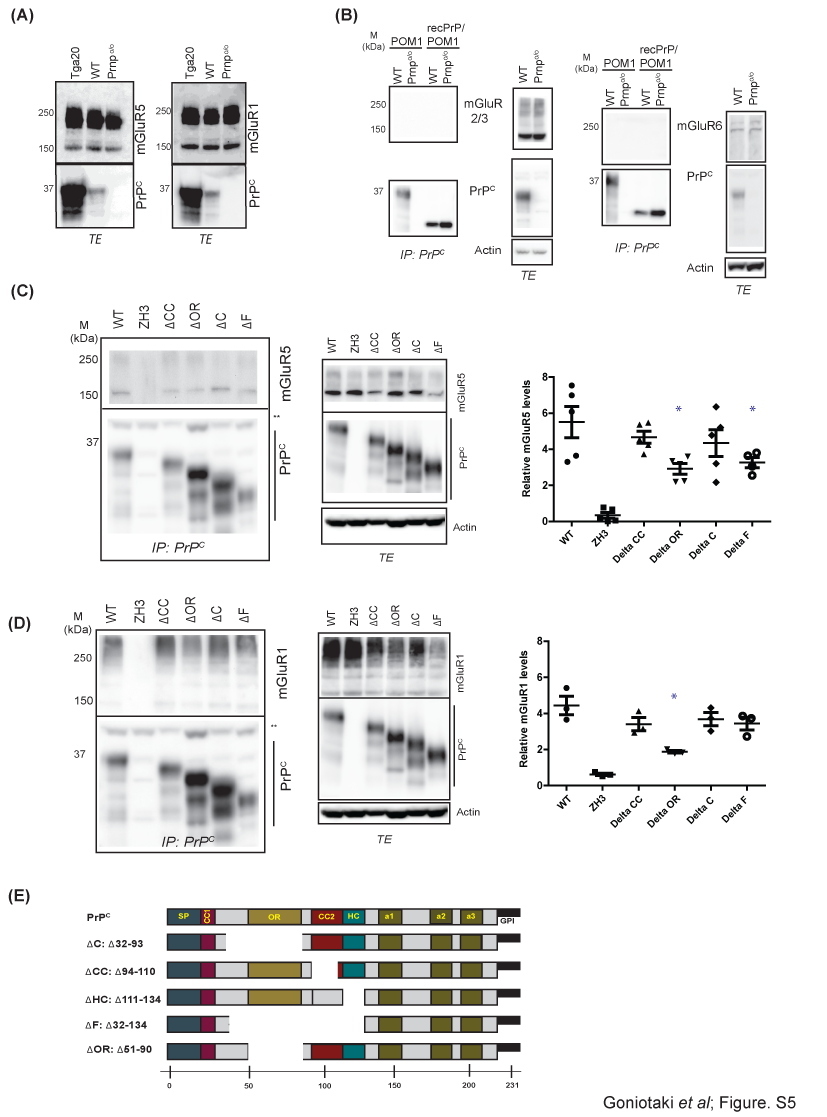

Supplement: S5 Fig — (A) Total brain extacts from wild-type (C57BL/6J), Tga20 and Prnpo/o mice was subjected to western blot analysis for the endogenous levels of mGluR5 and mGluR1. Expression of mGlur5/1 was similar in all the three mice model systems. (B) Brain homogenate from wild-type (C57BL/6J) and Prnpo/o mice was subjected to immunoprecipitation by POM1 followed by immunoblotting using polyclonal anti-mGluR2/3 and anti-mGluR6, or anti-PrPC antibodies. mGluR2/3 and mGluR6 did not coprecipitate with PrPC. Total brain extracts were in parallel subjected to Western blot analysis to control for endogenous levels of mGluR2/3 or 6 and PrPC. (C-D) Mapping the mGluR5 and mGluR1 interacting domains on PrPC. Brain homogenate from wild-type, Prnpo/o (ZH3) and amino proximal deletion mutants of PrPC was subjected to immunoprecipitation by POM1, followed by immunoblotting using polyclonal anti-mGluR5 (C) or anti-mGluR1 (D) and anti-PrPC antibodies. Deletions extending from residues 51–90 and 32–134, corresponding to the OR (octapeptide repeat region) and the flexible tail of PrPC, reduced the interaction with mGluR5, whereas deletions extending from residues 51 to 90, corresponding to the OR region of PrPC, decreased the interaction with mGluR1. Total brain extracts (TEs) were subjected to Western blot analysis to control for endogenous levels of mGluR5/1 and PrPC. Densitometric quantitation of mGluR1 or mGluR5 signal from the immunoprecipitation was normalized over the ration of Grm/Actin signal in TEs. Graphs represents mGluR1 or mGluR5 relative signal intensity; N = 3–5; One-way ANOVA followed by Tukey’s post-hoc test; n*: P<0.05. **: band corresponding to recombinant PrP. (E) Schematic representation of PrPC deletion mutants. Toxic POM1 antibody binds to a1-a3 helixes (residues 138–147; 204/208/212), innocuous POM2 antibody binds to octapeptide repeat (OR) region (residues 57–88), whereas POM3 antibody binds to residues 95–100 on PrPC. (TIF) [file ppat.1006733.s005.tif]

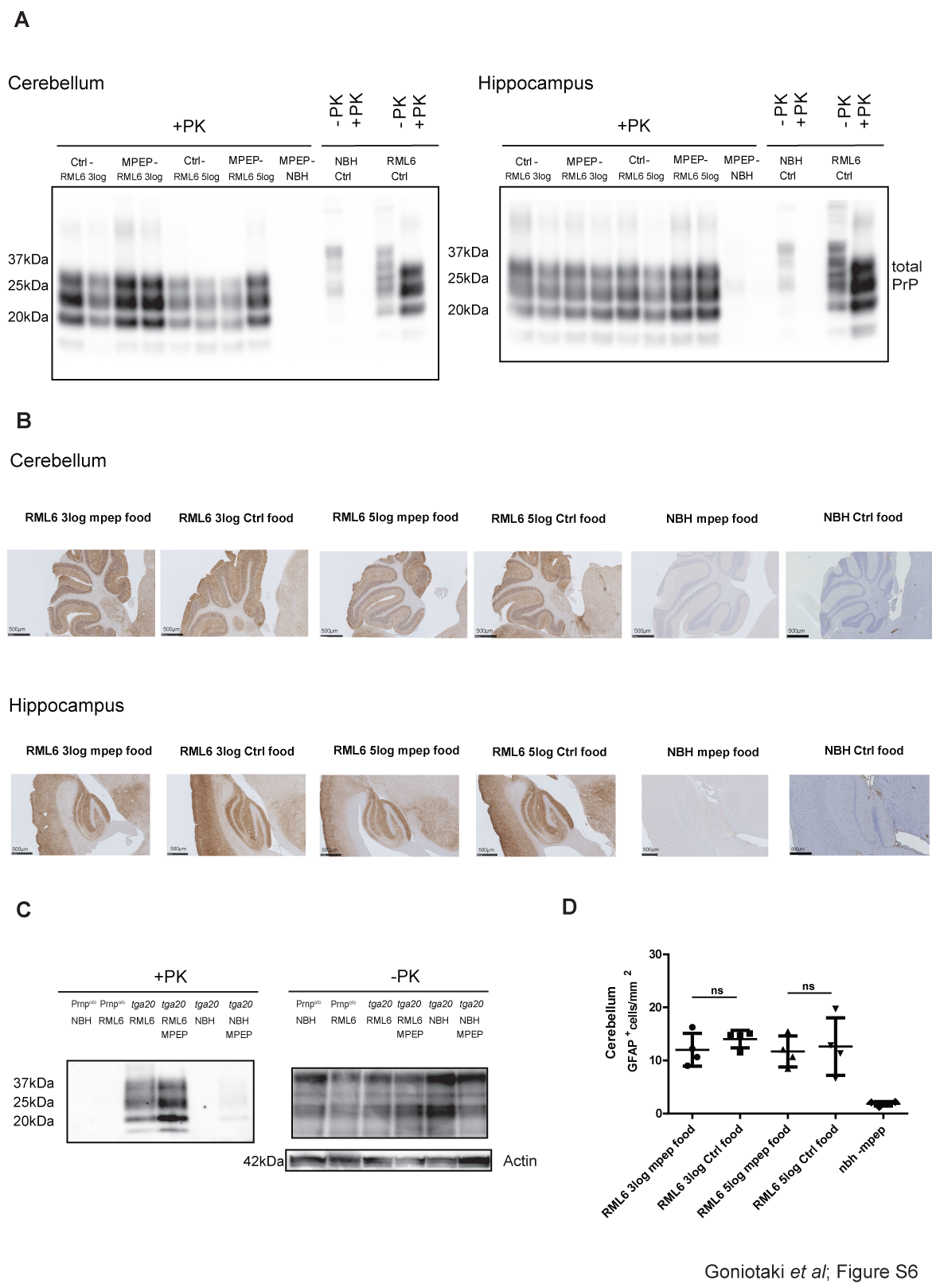

Supplement: S6 Fig — (A) Total PrP and PrPSc levels (detected by addition of proteinase K (PK)) in homogenates from different brain regions (hippocampus and cerebellum) of terminal C57BL/6J mice injected i.c. with NBH or RML6 prions and treated with control or MPEP-containing food respectively. Control NBH and RML6 samples, with or without addition of PK were run in parallel. (B) Representative images of SAF84-stained cerebellar and hippocampal sections from C57BL/6J mice injected i.c. with NBH or RML6 prions and treated with control or MPEP-containing food respectively. The levels of PrPSc (detected by SAF84 immunohistochemistry) are similar in brain sections from prion-infected mice treated with control or MPEP-containing food. (C) Total PrP and PrPSc levels (detected by addition of proteinase K (PK)) in homogenates from RML6 infected cerebellar slices prepared from tga20 or PrPo/o mice. Cerebellar slices infected with RML6 prions were also treated with MPEP according to the previously described protocol. Control NBH samples, with or without addition of PK were run in parallel. (D) Astrocyte proliferation was analyzed by immunohistochemistry with the GFAP antibody on cerebellar sections from C57BL/6J mice injected i.c. with NBH or RML6 prions and treated with control or MPEP-containing food respectively. Number of GFAP+ cells was quantified in the cerebellar granular layer (CGL). Dot blots represent mean ± SD GFAP expression, quantified as the percentage of the surface occupied by the GFAP staining over the total measured area; 10 regions of interest per slice, 4 slices per mouse and 4 mice per treatment group were used for quantification; two-way ANOVA followed by Bonferroni's post-hoc test. (TIF) [file ppat.1006733.s006.tif]

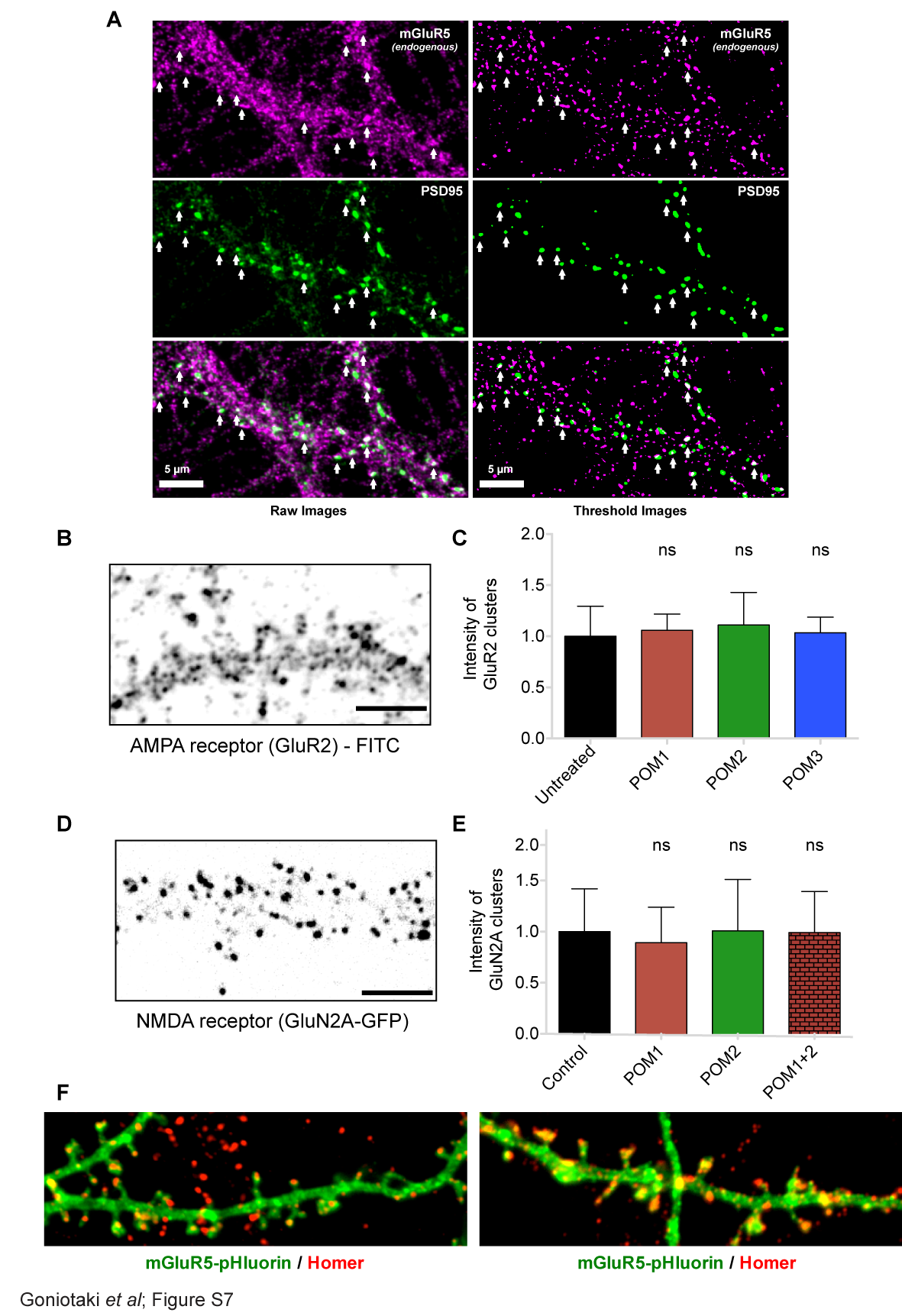

Supplement: S7 Fig — (A) Immunoreactivity of mGluR5s and PSD95 in cultured hippocampal neurons. Threshold images show the identified clusters. Arrow indicates that synaptic clusters co-localize with mGluR5 clusters. (B) Representative image (control condition) showing the immunoreactivity of GluR2 subunit of AMPA receptor following methanol fixation / permeabilization. Scale bar: 2 μm. (C) Quantification of the fluorescence intensity indicate that cluster size was not modified following POM antibodies application (One-way ANOVA with Dunnett’s post-hoc test relative to control; field of view (n): Control-22, POM1-22, POM2-22, POM3-22 from 2-independent experiments). (D) Representative image (control condition) showing the fluorescence of GluN2A-GFP subunit of NMDA receptor ~48 h after transfection and paraformaldehyde fixation. Scale bar: 2μm. (E) Quantification of fluorescence intensity indicate that the cluster size was not modified following POM antibodies application (One-way ANOVA with Dunnett’s post-hoc test relative to control; field of view (n): Control-22, POM1-22, POM2-22, POM3-20 from 2-independent experiments). (F) Representative images showing that the spines in mGluR5-SEP transfected neurons co-localize with the post-synaptic marker, Homer (which is also the scaffold of mGluR5s). (TIF) [file ppat.1006733.s007.tif]
