# Supplementary material for: Inhibition of group-I metabotropic glutamate receptors protects against prion toxicity
Source: PLoS Pathog. 2017 Nov 27;13(11):e1006733. doi: 10.1371/journal.ppat.1006733 (PMC5720820; doi:10.1371/journal.ppat.1006733)
Supplement: S1 Table — MPEP levels were assessed in blood and brain of mice at two circadian points within a day (light/dark cycle). The brain-to-plasma ratios were calculated based on this analysis and is represented in the tables. The upper table contains the levels of MPEP in brain and the lower table contains the levels of MPEP in blood. (PDF) [file ppat.1006733.s008.pdf]

Table S1. MPEP values (diurnal measurements) in brain and blood samples

Brain levels of CGP79775A/MPEP (Mouse PKPD MPEP Prof Aguzzi Coll)

brain	ng/g	pmol/g
LLOQ:	2.4	12.4
ULOQ:	7'500.0	38'819.9

Sample Number	Sample Identifier	Amount [ng/g]	Amount [pmol/g]	Remarks	Sample Collection Time	Food
11	H922_S011_brn_po_mm1_30_MPEP_0H	12.3	63.4		LC	MPEP
12	H922_S012_brn_po_mm2_30_MPEP_0H	4.3	22.4		LC	MPEP
13	H922_S013_brn_po_mm3_30_MPEP_0H	62.5	323.7		LC	MPEP
14	H922_S014_brn_po_mm4_30_MPEP_0H			BQL	LC	MPEP
15	H922_S015_brn_po_mm5_30_MPEP_0H	36.2	187.5		LC	MPEP
16	H922_S016_brn_po_mm6_30_MPEP_0H			BQL	LC	MPEP
17	H922_S017_brn_po_mm7_0_MPEP_0H			BQL	LC	CONTROL
18	H922_S018_brn_po_mm8_0_MPEP_0H			BQL	LC	CONTROL
19	H922_S019_brn_po_mm9_0_MPEP_0H			BQL	LC	CONTROL
20	H922_S020_brn_po_mm10_0_MPEP_0H			BQL	LC	CONTROL
31	H922_S031_brn_po_mm11_30_MPEP_0H	75.8	392.3		DC	MPEP
32	H922_S032_brn_po_mm12_30_MPEP_0H	28.8	149.2		DC	MPEP
33	H922_S033_brn_po_mm13_30_MPEP_0H	22.3	115.4		DC	MPEP
34	H922_S034_brn_po_mm14_30_MPEP_0H	55.2	285.6		DC	MPEP
35	H922_S035_brn_po_mm15_30_MPEP_0H	67.5	349.4		DC	MPEP
36	H922_S036_brn_po_mm16_30_MPEP_0H	60.6	313.5		DC	MPEP
37	H922_S037_brn_po_mm17_0_MPEP_0H			BQL	DC	CONTROL
38	H922_S038_brn_po_mm18_0_MPEP_0H			BQL	DC	CONTROL
39	H922_S039_brn_po_mm19_0_MPEP_0H			BQL	DC	CONTROL
40	H922_S040_brn_po_mm20_0_MPEP_0H			BQL	DC	CONTROL

LC: light cycle (inactive state)
DC: dark cycle (active state)

Blood levels of CGP79775A/MPEP (Mouse PKPD MPEP Prof Aguzzi Coll)

blood	ng/mL	nM
LLOQ:	0.8	4.1
ULOQ:	2'500.0	12'940.0

Sample Number	Sample Identifier	Amount [ng/ml]	Amount [nM]	Remarks	Sample Collection Time	Food
1	H922_S001_bld_po_mm1_30_MPEP_0H	10.9	56.2		LC	MPEP
2	H922_S002_bld_po_mm2_30_MPEP_0H	4.5	23.2		LC	MPEP
3	H922_S003_bld_po_mm3_30_MPEP_0H	64.9	335.9		LC	MPEP
4	H922_S004_bld_po_mm4_30_MPEP_0H	1.9	9.6		LC	MPEP
5	H922_S005_bld_po_mm5_30_MPEP_0H	38.7	200.4		LC	MPEP
6	H922_S006_bld_po_mm6_30_MPEP_0H	0.9	4.7		LC	MPEP
7	H922_S007_bld_po_mm7_0_MPEP_0H			BQL	LC	CONTROL
8	H922_S008_bld_po_mm8_0_MPEP_0H			BQL	LC	CONTROL
9	H922_S009_bld_po_mm9_0_MPEP_0H			BQL	LC	CONTROL
10	H922_S010_bld_po_mm10_0_MPEP_0H			BQL	LC	CONTROL
21	H922_S021_bld_po_mm11_30_MPEP_0H	38.1	197.3		DC	MPEP
22	H922_S022_bld_po_mm12_30_MPEP_0H	23.1	119.8		DC	MPEP
23	H922_S023_bld_po_mm13_30_MPEP_0H	23.1	119.7		DC	MPEP
24	H922_S024_bld_po_mm14_30_MPEP_0H	35.3	182.6		DC	MPEP
25	H922_S025_bld_po_mm15_30_MPEP_0H	38.8	200.6		DC	MPEP
26	H922_S026_bld_po_mm16_30_MPEP_0H	54.7	282.9		DC	MPEP
27	H922_S027_bld_po_mm17_0_MPEP_0H			BQL	DC	CONTROL
28	H922_S028_bld_po_mm18_0_MPEP_0H			BQL	DC	CONTROL
29	H922_S029_bld_po_mm19_0_MPEP_0H			BQL	DC	CONTROL
30	H922_S030_bld_po_mm20_0_MPEP_0H			BQL	DC	CONTROL
